# Supplementary material for: Quantifying intervals to diagnosis in myeloma: a systematic review and meta-analysis
Source: BMJ Open. 2018 Jun 22;8(6):e019758. doi: 10.1136/bmjopen-2017-019758 (PMC6020959; doi:10.1136/bmjopen-2017-019758)

A1: Systematic review protocol

Title:

Delays observed in the pathway leading to the diagnosis of multiple myeloma - a systematic review

Background

Multiple myeloma is one of the most common haematological malignancies, with more than 4000 cases diagnosed annually. It is considered as one of the most difficult cancers to diagnose due the very non-specific nature of symptoms which might include bone pain, fatigue, dyspnoea, weight loss, repeated infections etc. 51% of the symptomatic myeloma patients have to visit their GP at least 3 times before they get a confirmed diagnosis of the disease and 38% of the patients are identified through emergency admissions (compared to 23% for the rest of the cancers)1. A study has suggested that a delayed diagnosis had a significant effect on disease-free survival 2 so an earlier diagnosis of the disease could potentially lead to fewer complications, a better prognosis and a better quality of life. The aim of this systematic review is to examine the published literature for diagnostic delay in multiple myeloma across the diagnostic pathway.

1. Lyratzopoulos, G., Neal, R. D., Barbiere, J. M., Rubin, G. P. & Abel, G. a. Variation in number of general practitioner consultations before hospital referral for cancer: Findings from the 2010 National Cancer Patient Experience Survey in England. Lancet Oncol. 13, 353–365 (2012).

2. Kariyawasan, C. C., Hughes, D. a., Jayatillake, M. M. & Mehta, a. B. Multiple myeloma: causes and consequences of delay in diagnosis. Qjm 100, 635–640 (2007).

Review questions/objectives:

Quantification of the time intervals that multiple myeloma patients experience from first symptom to confirmation of diagnosis. The time interval will include the patient intervals (onset of first symptom to help seeking), primary care interval (from first presentation to primary care until first referral), secondary care interval (first referral to diagnosis), diagnostic interval (first presentation to diagnosis) and the total interval (onset of symptoms to diagnosis).

Searches and eligibility criteria

A systematic literature search will be performed in MEDLINE and EMBASE. All articles that are quantifying any of the intervals mentioned above will be included. Articles on non-adults (<18 years) and on the asymptomatic form of the disease will be excluded.

Type of studies to be included:

Cross-sectional surveys, prospective patient studies and retrospective analysis of medical records which give a numerical measure of diagnostic delay in multiple myeloma. Only full text articles will be included in the review. Conference abstracts will be excluded.

Intervention(s)/exposure(s)

None

Comparator(s)/control

None

Primary outcomes

Clinical diagnostic interval (first presentation to final diagnosis)

Patient interval (symptom onset to first presentation)

Primary care interval (first presentation to first referral)

Secondary care interval (first referral to final diagnosis)

Total interval (symptom onset to final diagnosis)

Data extraction (selection and coding)

Two reviewers (CK and LA) will extract the data from the included studies in pre-specified forms. Disagreements will be resolved by consulting a third reviewer (AVB or JO). Variables that are going to be extracted from the papers include: Author, Year of study, Country, Study Design, sample size, initial symptoms and descriptive statistics for the interval under investigation (mean, Sd, median and IQR).

Risk of bias (quality assessment)

The methodological quality of the papers will be assessed by using the Aarhus checklist

Strategy for data synthesis

Mean, median and interquartile range will be extracted for the analysis but since time duration is usually not a normally distributed variable the median and IQR will be preferred. Delays that are reported in months will be transformed into days. In papers where the number of consultations is reported instead of a numeric value of the delay we will contact authors otherwise these papers will be excluded. We will try to combine our estimates in order to get an overall estimate of the delay observed for each interval.

Analysis of subgroups or subsets

Subgroup analysis will be conducted comparing studies with the highest risk of bias versus the rest.

Dissemination plans

This review will be published in a peer-reviewed journal and presented in relevant conferences

A2: Search strategy

1. Multiple Myeloma/
2. myeloma*.ti,ab.
3. 1 or 2
4. (time adj4 diagnos$).ti,ab.
5. (time adj4 consult$).ti,ab.
6. (time adj4 refer$).ti,ab.
7. (time adj4 present$).ti,ab.
8. 4 or 5 or 6 or 7
9. (delay$ adj4 diagnos$).ti,ab.
10. (delay$ adj4 consult$).ti,ab.
11. (delay$ adj4 refer$).ti,ab.
12. (delay$ adj4 present$).ti,ab.
13. (delay$ adj4 seek$).ti,ab.
14. (delay$ adj4 detect*).ti,ab.
15. 9 or 10 or 11 or 12 or 13 or 14
16. (interval adj4 consult$).ti,ab.
17. (interval adj4 consult$).ti,ab.
18. (interval adj4 refer$).ti,ab.
19. (interval adj4 present$).ti,ab.
20. 16 or 17 or 18 or 19
21. (late adj4 diagnosis).ti,ab.
22. (late adj4 detect*).ti,ab.
23. (late adj4 present$).ti,ab.
24. 21 or 22 or 23
25. diagnos$ delay$.ti,ab.
26. early diagnos$.ti,ab.
27. 8 or 15 or 20 or 24 or 25 or 26
28. 3 and 27

A3: Risk of bias summary

Legend: No (red) · Yes (green) · Not applicable (yellow)

	1	2	3	4	5	6	7	8	9	10	11	12	13	14	15	16	17	18	19	20
Friese et al. 2009	Yes	Yes	Not applicable	Not applicable	No	Not applicable	No	Yes	Yes	No	Not applicable	Not applicable	Not applicable	Not applicable	Not applicable	Not applicable	Not applicable	Not applicable	Not applicable	Yes
Howell et al. 2013	Yes	Yes	No	Yes	Yes	Not applicable	No	Yes	Yes	Yes	No	Yes	No	No	Yes	Yes	No	Yes	Not applicable	Not applicable
Lyratzopoulos et al. 2013	Yes	Yes	Not applicable	Not applicable	Yes	Yes	No	Yes	Yes	Yes	Not applicable	Not applicable	Not applicable	Not applicable	Not applicable	Not applicable	Not applicable	Not applicable	Not applicable	Yes
Varga et al. 2014	Yes	No	Not applicable	Not applicable	No	Not applicable	No	Yes	Yes	No	Not applicable	Not applicable	Not applicable	Not applicable	Not applicable	Not applicable	Not applicable	Not applicable	No	Not applicable
Neal et al. 2014	Yes	Yes	Not applicable	Not applicable	Yes	Not applicable	Yes	Yes	Yes	Yes	Not applicable	Not applicable	Not applicable	Not applicable	Not applicable	Not applicable	Not applicable	Not applicable	Not applicable	Yes
Din et al. 2015	Yes	Yes	Not applicable	Not applicable	Yes	Not applicable	Yes	Yes	Yes	Yes	Not applicable	Not applicable	Not applicable	Not applicable	Not applicable	Not applicable	Not applicable	Not applicable	Not applicable	Yes
Lyratzopoulos et al. 2015	Yes	Yes	Yes	Yes	Yes	Not applicable	No	Yes	Yes	Yes	Not applicable	Not applicable	Not applicable	Not applicable	Not applicable	Not applicable	Not applicable	Not applicable	Not applicable	Yes
Goldscbmidt et al. 2016	Yes	Yes	Not applicable	Not applicable	Yes	Not applicable	No	Yes	Yes	No	Not applicable	Not applicable	Not applicable	Not applicable	Not applicable	Not applicable	Not applicable	Not applicable	Yes	Not applicable
Swann et al. 2017	Yes	Yes	Not applicable	Not applicable	Yes	Yes	No	Yes	Yes	Yes	Not applicable	Not applicable	Not applicable	Not applicable	Not applicable	Not applicable	Not applicable	Not applicable	Yes	Not applicable

A4: Risk of bias graph

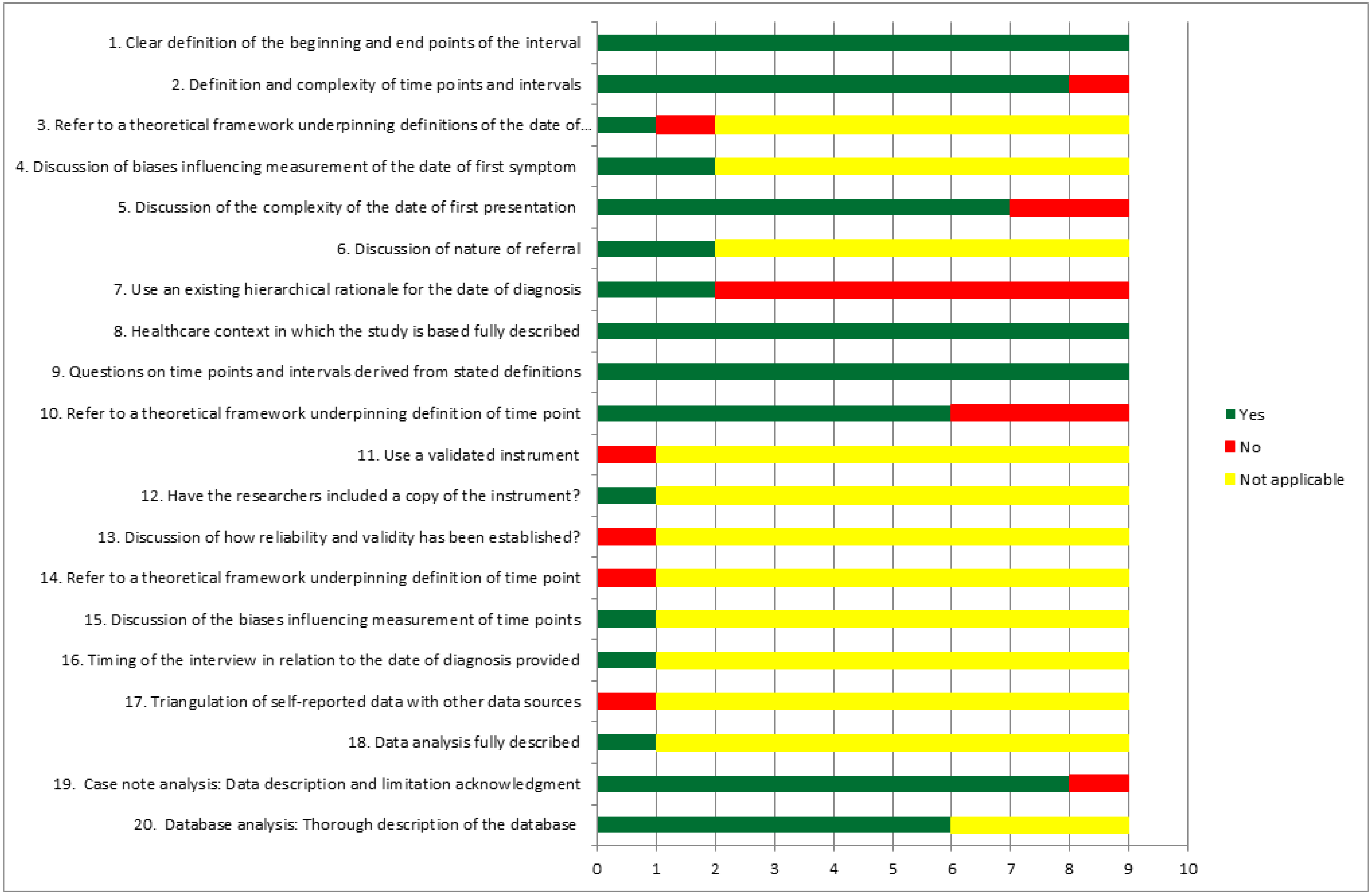

A5: Random effects meta-analysis combining means. For studies that were not reporting means they were approximated using the median and interquartile range.

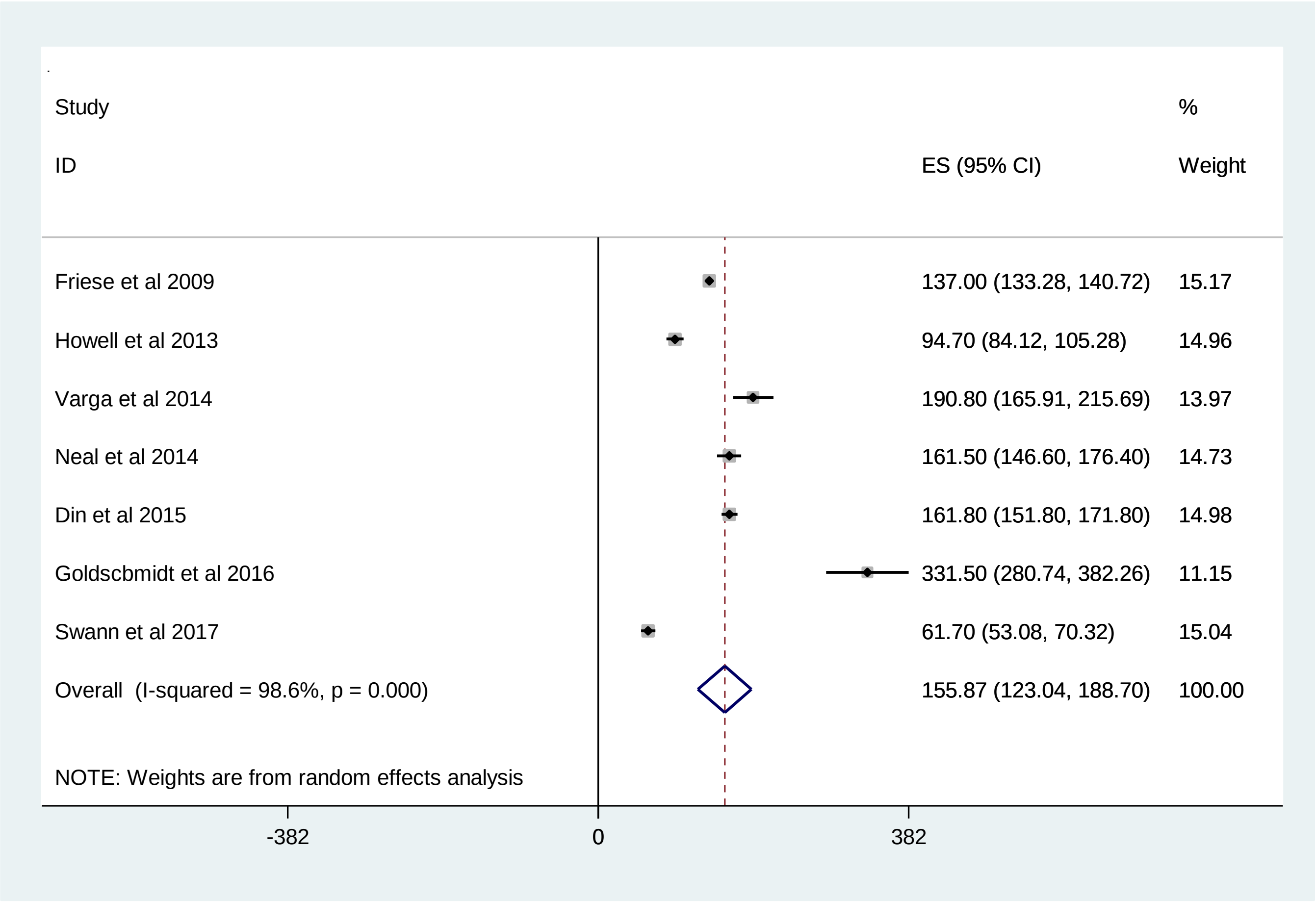

Supplement: Supplementary file 1 [file bmjopen-2017-019758supp001.pdf]
