# Supplementary material for: Quantifying intervals to diagnosis in myeloma: a systematic review and meta-analysis
Source: BMJ Open. 2018 Jun 22;8(6):e019758. doi: 10.1136/bmjopen-2017-019758 (PMC6020959; doi:10.1136/bmjopen-2017-019758)
Supplement: Author's manuscript [file bmjopen-2017-019758.draft_revisions.pdf]

BMJ Open

BMJ Open is committed to open peer review. As part of this commitment we make the peer review history of every article we publish publicly available.

When an article is published we post the peer reviewers' comments and the authors' responses online. We also post the versions of the paper that were used during peer review. These are the versions that the peer review comments apply to.

The versions of the paper that follow are the versions that were submitted during the peer review process. They are not the versions of record or the final published versions. They should not be cited or distributed as the published version of this manuscript.

BMJ Open is an open access journal and the full, final, typeset and author-corrected version of record of the manuscript is available on our site with no access controls, subscription charges or pay-per-view fees (http://bmjopen.bmj.com).

If you have any questions on BMJ Open's open peer review process please email info.bmjopen@bmj.com

Quantifying diagnostic intervals in myeloma: a systematic review and meta-analysis

Journal:	*BMJ Open*
Manuscript ID	bmjopen-2017-019758
Article Type:	Research
Date Submitted by the Author:	25-Sep-2017

Complete List of Authors:	Koshiaris, Constantinos; University of Oxford Department of Primary Care Health Sciences<br>Oke, Jason; University of Oxford, Nuffiled Department of Primary Care Health Sciences<br>Abel, Lucy; University of Oxford, Nuffield Department of Primary Care Health Sciences<br>Nicholson, Brian; University of Oxford, Nuffield Dept Primary Care Health Sciences<br>Ramasamy, Karthik ; Churchill Hospital, Department of Haematology, Oxford University Hospitals NHS Trust<br>van den Bruel, Ann; University of Oxford, Dept of Primary Health Care

**Primary Subject Heading**:	Haematology (incl blood transfusion)
Secondary Subject Heading:	Health services research
Keywords:	Myeloma < HAEMATOLOGY, time to diagnosis, early diagnosis, PRIMARY CARE, systematic review, delays

SCHOLARONE™
Manuscripts

Quantifying diagnostic intervals in myeloma: a systematic review and meta-analysis

Constantinos Koshiaris[1] MSc, Jason Oke[1] PhD, Lucy Abel[1] MSc, Brian D Nicholson[1] MRCGP, Karthik Ramasamy[2 3] PHD, Ann Van den Bruel PHD[1]

[1]Nuffield Department of Primary Care Health Sciences, University of Oxford, Radcliffe Primary Care Building, Radcliffe Observatory Quarter, Woodstock Road, Oxford OX2 6GG

[2] Department of Haematology, Oxford University Hospitals NHS Trust, Old Road, Headington, Oxford OX3 7LE

[3] NIHR BRC Blood Theme, Oxford

Corresponding author:

Mr. Constantinos Koshiaris (constantinos.koshiaris@phc.ox.ac.uk)

Telephone number: 01865 289317

Nuffield Department of Primary Care Health Sciences, University of Oxford, Radcliffe Primary Care Building, Radcliffe Observatory Quarter, Woodstock Road, Oxford OX2 6GG

Word count: 3060

Key words: multiple myeloma, time to diagnosis, early diagnosis, primary care, systematic review, delays

For peer review only - http://bmjopen.bmj.com/site/about/guidelines.xhtml

ABSTRACT

Objectives

To quantify the duration of each step of the diagnostic pathway for multiple myeloma patients from symptom onset to confirmation of diagnosis

Design

Systematic review and meta-analysis

Data sources and selection criteria

The MEDLINE and EMBASE databases were searched up until February 2016 to identify articles which reported time intervals from onset of symptoms to diagnosis. Articles focusing on children or adolescents and on the asymptomatic form of the disease (monoclonal gammopathies and smouldering myeloma) were excluded.

Data collection and data analysis

Data were extracted independently by two reviewers. Weighted estimates of the median and interquartile range were calculated.

Main results

Seven studies were included. The patient interval has a median of 26.3 days (IQR: 1 to 98, n=465, 2 studies). Subsequently, the primary care interval is 21 days (IQR: 5 to 55, n=176, 1 study), the diagnostic interval 106 days (IQR: 34 to 247, n=5086, 5 studies) and the total interval 163 days (IQR: 84 to 306, n=341, 1 study). No studies were describing the secondary care interval; inference from the other studies suggests it might be between 85 and 142 days

Conclusion

The review demonstrates that there is scope for significant reductions in the time to myeloma diagnosis. At present, many patients experience a diagnostic interval longer than 3 months until diagnosis is confirmed. It is possible that the longest duration is in secondary care,

For peer review only - http://bmjopen.bmj.com/site/about/guidelines.xhtml

which might be driven by the type and urgency of referral but further research is needed to confirm this.

Review registration

Not available. Protocol available from authors

Strengths and limitations of this study

Strengths

- First systematic review that quantified the whole diagnostic pathway for multiple myeloma patients including the different intervals in each step of the pathway
- A comprehensive search strategy with no design restrictions to capture all available information
- Use of all available information including the interquartile range rather than just focusing on measures of central tendency like the mean and the median

Limitations

- No universally accepted methods for formal meta analysis of median and interquartile range
- Limited number of studies reporting most secondary outcomes

For peer review only - http://bmjopen.bmj.com/site/about/guidelines.xhtml

INTRODUCTION

Myeloma is a haematological malignancy characterised by uncontrolled plasma cell production in the bone marrow. It was the 17th most common cancer in the UK in 2013 accounting for 2% of all new cancer cases. Currently there are more than 17500 myeloma patients in the UK with approximately 5500 cases being diagnosed every year[1,2]. It is a cancer that mainly affects the elderly population with 59% of the patients being diagnosed over the age of 70[2] and with a 5 year survival of 47%[3].

It is considered one of the hardest cancers to suspect in primary care. Symptoms of myeloma are very common in other conditions as well, such as back pain, bone pain, fatigue and repeated infections[4]. This in combination with the fact that myeloma is a very rare condition in primary care results in very low predictive values for individual symptoms. For example, primary care patients with back pain, which is one of the most common myeloma symptoms, only have a 0.1% risk of myeloma[5]. By comparison, patients with rectal bleeding have a 2.4% risk of colorectal cancer[6].

As a result, half of symptomatic myeloma patients have three or more consultations in primary care before they are referred to specialist care which is more than in any other cancer[7]. Attributing symptoms to comorbidities further prolongs the diagnostic process, which is particularly relevant in this older age group [8,9].

Delays in diagnosing myeloma allow complications to develop (end organ damage), such as pathological fractures, irreversible renal failure and in some cases spinal cord compression[10–12]. These are considered medical emergencies in their own right and limit the opportunity for applying effective treatment[13]. A delayed diagnosis is also linked with higher cancer stage[14,15] which is in turn associated with poorer survival[16]. Patients with

For peer review only - http://bmjopen.bmj.com/site/about/guidelines.xhtml

longer diagnostic intervals also experience shorter disease free survival and more complications from treatment[14].

Quantifying the time-intervals leading up to diagnosis is important as it will inform future interventions that aim to shorten the diagnostic process. The aim of this systematic review was to quantify each step of the diagnostic pathway to myeloma diagnosis and identify where to focus efforts to reduce diagnostic delay.

METHODS

A protocol is available on request from the authors. A copy of the search strategy can be seen in the appendix. We searched EMBASE and MEDLINE until February 2016 for studies that quantified any or all of the following five intervals[17]: the patient interval (from symptom onset to first consultation); the primary care interval (from first consultation for that symptom to referral to secondary care); the secondary care interval (from referral to diagnosis of myeloma); the diagnostic interval (from first consultation to diagnosis); and the total interval (from symptom onset to diagnosis) (Figure 1). We included any study designs that quantified at least one of the intervals mentioned above in days or months. Studies reporting the length of an interval only in number of consultations or referrals were excluded as were studies focusing on children or adolescents (< 18 years) and on the asymptomatic forms of the disease (monoclonal gammopathies and smouldering myeloma). We included papers with an abstract in English but did not exclude full text articles based on language. Two reviewers (CK/LA) selected papers for inclusion using the criteria listed above, on title and abstracts first and on full text second. Disagreements were resolved through discussion with a third reviewer (JO/AVB).

Data extraction

For peer review only - http://bmjopen.bmj.com/site/about/guidelines.xhtml

Two reviewers (CK/LA) independently extracted data from the included studies into a pre-defined spreadsheet. Study characteristics including author, year of publication, country of data collection, type of study, myeloma related symptoms and sample size were extracted, as well as descriptive statistics including median, interquartile range, range, mean and the standard deviation (SD) for each interval. Authors were contacted if data were not available or not in the appropriate format for extraction (i.e. categorical rather than continuous).

Risk of bias assessment

The risk of bias was assessed by two independent researchers (CK/LA) using the Aarhus checklist[18]. The Aarhus checklist is a 20 item tool designed to help researchers design and evaluate studies on early diagnosis of cancer and examines studies in terms of acknowledgment of the different biases influencing time point measurement and interval definition, questionnaire validation and data collection in patient reported data and analysis of case-note audits and databases. We assessed clinical heterogeneity of the included studies in terms of time-points, interval and symptom definitions.

Analysis

In the context of illness duration, intervals are usually not normally distributed, therefore we used the median and interquartile-range (IQR) to summarise the data. We present the 25th, 50th and 75th percentiles for all intervals. When more than one studies were available, we pooled the results by calculating a weighted mean for each percentile where the weight was obtained by dividing the sample size in each study with the total numbers of patients. We also fitted a distribution through the 3 weighted percentile estimates where appropriate in order to try and generate the shape of the distribution of the interval under investigation. We chose the lognormal distribution as time intervals are usually skewed to the right[4]. A pre-specified sensitivity analysis was conducted by excluding the study with the higher risk of bias. The

For peer review only - http://bmjopen.bmj.com/site/about/guidelines.xhtml

sensitivity analysis was conducted only for the diagnostic interval as the rest of the outcomes were reported by only 1 or 2 papers.

RESULTS

We identified 2816 citations from the EMBASE and MEDLINE searches. After removal of conference abstracts and duplicates, we screened 1036 titles and abstracts and seven studies were included in the final analysis (Figure 2).

Study Characteristics

A summary of all the included papers is provided in table 1. Studies were published between 2009 and 2015 and the sample size ranged from 124 to 3831 patients. Four studies reported diagnostic intervals in various cancers; two reported only myeloma; and one for haematological malignancies.

Five studies were conducted in the UK, one in the USA and one in Hungary. Two UK studies used data from two separate CPRD cohorts[19,20], a database of routinely collected electronic primary care records. Two other UK studies used data from the English National Audit of Cancer Diagnosis in Primary Care 2009-2010[21]: we extracted primary care interval data from the larger study and the patient interval from the smaller study[22,23]. The last UK study was a patient survey on patients diagnosed with haematological malignancies[24]. The study conducted in Hungary analysed data collected from patients treated in a haematology centre and the study conducted in the USA analysed a retrospective database collected from the SEER program[8,25].

Definition of diagnostic intervals

There was substantial heterogeneity in the symptoms and time points used to define each interval (table 3). In total, 19 different symptoms were used to define the start of myeloma

For peer review only - http://bmjopen.bmj.com/site/about/guidelines.xhtml

but studies varied greatly regarding which symptom (or symptoms) was used, ranging from 3 to a maximum of 7 symptoms. Two studies did not report the starting symptoms[22,23]. Also some studies included multiple symptoms in more general categories[19,20,24]. For example, Howell et al used a general pain category which included musculoskeletal, abdominal, chest and other type of pains while the two CPRD studies included multiple musculoskeletal symptoms under a general bone pain category[19,20].

The start of the measuring period was defined as the date of onset of the first symptom or the date of first presentation for a myeloma related symptom depending on whether the studies were investigating the patient interval, the diagnostic or both. Out of seven studies, three identified the first symptom within the year preceding diagnosis, one at three years, two at two years before diagnosis and one study used patient reported dates.

Risk of Bias

Most of the studies included in the analysis had a low risk of bias (Appendix). All studies clearly defined the start and end point of the intervals and in most cases there was an adequate description of the databases along with the strengths, limitations and biases arising from the definitions of the different intervals and time point. Only one study did not mention the different limitations and biases arising from the study design and the choice of definitions for time points and intervals[25]. Most common sources of bias that were described included recall bias for studies that were using patient reported data and misclassification bias for studies that were using databases like CPRD. Most studies used a theoretical framework to define each interval usually the one reported by Olesen et al[17] or the Aarhus statement[18]. The category with the higher risk of bias was the use of a hierarchical rationale to determine the date of diagnosis i.e. date of first histological confirmation of the malignancy or date of admission to the hospital for example. Most studies mentioned how the date of diagnosis was

For peer review only - http://bmjopen.bmj.com/site/about/guidelines.xhtml

obtained but there was no adequate description on how the choice of a particular definition can affect the diagnostic pathway.

Quantifying intervals

Five papers reported the diagnostic interval[8,19,20,24,25], two the patient interval[22,24], one the primary care interval[23], and one the total interval[24]. No studies reported the secondary care interval. The length of the different intervals can be seen in table 2 and the fitted log normal distributions in figure 3 along with the parameters used to fit them.

For the diagnostic interval, the pooled weighted mean of the 50th percentile is 105.8 days (IQR: 33.7 - 247.1, n=5086). Removing the study with the largest risk of bias[25] based on the Aarhus statement checklist did not alter the results (105.2 days, IQR: 31.6-247.7).

The pooled weighted mean of the 50th percentile of the patient interval is 26.3 days (IQR: 0.7-97.7, n=465). The primary care interval was reported by only one study[23] with a median of 21 days (IQR: 5-55, n=176) and the total interval was also reported by one study[24] with a median of 163 days (IQR: 84-306, n=341).

No study reported the secondary care interval but the length of the secondary care interval can be inferred by subtracting the median length of the primary care interval from the diagnostic interval or the total interval which suggests that the median length of the secondary care interval can range from 85 day to 142 days depending on whether we use the diagnostic or the total interval.

DISCUSSION

Our results show that myeloma patients experience symptoms for a median of approximately one month before seeking help and 25% of patients wait for more than three months (98 days). After attending primary care with symptoms, the median time to diagnosis is 106 days

For peer review only - http://bmjopen.bmj.com/site/about/guidelines.xhtml

(IQR: 34-247) with 25% of patients waiting longer than eight months. However, the time between referral to secondary care and diagnosis can range from 85 to 142 days depending on whether we use the total or the diagnostic interval to infer the secondary care interval and it suggests that secondary care interval can be four to seven times greater than the primary care interval.

Strengths and weaknesses

We used a comprehensive search strategy with no design restrictions to capture all available information. We also did not focus on measures of central tendency like the mean and median but also included the 25th and 75th percentiles to which is particularly important since time interval data are skewed to the right which means that some people experience much longer intervals than what the measures of central tendency suggest.

There are currently no universally accepted formal methods to perform meta-analysis of medians and interquartile ranges. To overcome this, we combined estimates of the percentiles after weighting them based on the sample size: a method equivalent to a fixed effects meta-analysis. We were not able to produce a confidence interval around the median and interquartile range as these are not usually measures that are reported by studies thus we present only the point estimate of each percentile. Also we were not able to estimate measures of statistical heterogeneity.

Furthermore we identified only one study reporting the primary care interval and no studies for the secondary care interval so any conclusions about these intervals should be interpreted with caution.

Variability in definitions of time points

For peer review only

In order to compute intervals, the definition of the beginning and the end of the interval is crucial. There was variability in how studies defined starting points, especially for the first symptom and the first presentation to healthcare, using medical records or patient recall. Studies that use patient reported outcomes tend to suffer from recall bias which might lead to overestimation or underestimation of the different intervals while studies using medical records tend to suffer from loss to follow up and misclassification.

For studies that were using electronic health records there was no agreement on when exactly myeloma starts to manifest. The time used to detect related symptoms prior to diagnosis spanned from one to three years. Although most studies used one year before diagnosis, there is some evidence to suggest that symptoms might be present for more than one year[26] which may have led to an underestimation of intervals in these studies. On the other hand, the more you extend the symptom period the more likely you are to detect symptoms that are unrelated to myeloma which leads to the overestimation of the length of the intervals, especially with symptoms that are so aspecific such as back pain. In order to explore this we conducted a sensitivity analysis where we estimated the length of the diagnostic interval by stratifying according to the time used to define the presenting symptoms (one year back vs. three years back) which resulted in similar results (one year back: 107 (31-254) vs. three years back: 125 (88-230)). This could be affected thought by the fact that we had only one study going back up to three years with a small sample size. The rest of the studies that defined the presenting symptoms within two years before diagnosis did not investigate the diagnostic interval.

Findings compared to existing research

Our estimate for the patient interval is in-line with the findings of another study[27] which reported that 15% of myeloma patients wait more than three months before they go to the

For peer review only - http://bmjopen.bmj.com/site/about/guidelines.xhtml

doctor. This study explained this delay in terms of patients' lack of understanding of the seriousness of their symptoms because of their non-specific nature.

The diagnostic interval, which takes place in health care and could potentially be amenable to improvement, is longer for myeloma than for many other cancers. In breast cancer for example, the median diagnostic interval for symptomatic patients is reported to be 14 days[28], approximately seven and a half times shorter than our estimated median diagnostic interval for myeloma. Other cancers with a similarly long median diagnostic interval also have non-specific clinical presentations, such as lung cancer which has a diagnostic interval of 88 days[28] and leukaemia with a median of 102 days[19].

Implications for clinical practice

Trying to reduce intervals matters because longer intervals may be associated with more advanced disease[29]; the National Audit of Cancer diagnosis in Primary Care (2010-2011) reported that 22% of myeloma patients had a metastatic disease at diagnosis compared to 8.7% of breast cancer patients. It has also been reported by the Myeloma Patient Experience Report 2016 that more myeloma patients felt their health got worse while waiting to see a specialist than other cancer patients[30].

The primary care interval may be influenced by time spent waiting for the results of further investigations prior to referral[31]. Although investigations such as blood tests may prolong the primary care interval, patients may still benefit if they result in better targeted referrals, reducing the secondary care interval by avoiding an inappropriate referral which potentially can take more time than the time it takes for a blood result to come back.

Implications for further research

For peer review only - http://bmjopen.bmj.com/site/about/guidelines.xhtml

Although we were not able to estimate the secondary care interval directly, it is reasonable to believe that it is longer than the primary care interval. As in other cancers[32], suboptimal referrals, i.e. to the wrong specialty or with an insufficient level of urgency, prolong the secondary care interval. The choice of referral route has been shown to be a strong predictor of the length of the diagnostic interval[33]. Future studies should not only estimate the duration of primary and secondary care intervals, but also investigate the impact of one setting on the other.

Also it is still not clear how long before diagnosis myeloma symptoms start to occur. In lung cancer, studies on symptom lead time (the time between symptoms attributable to cancer and diagnosis) show that symptom incidence increases considerably 6 months before diagnosis[34] but no such study has been conducted for myeloma.

CONCLUSION

Our work suggests that there is potential for meaningful reductions in the time to diagnosis which could improve patient outcomes. Although the time from referral to secondary care to diagnosis appears to be the longest, we believe that shortening the secondary care interval could also be in the hands of the general practitioner as they choose the speciality and urgency of referral. More research is required to examine the myeloma patient pathway in more detail, including a more detailed breakdown of referral patterns in myeloma patients in terms of speciality and urgency. More decision making tools should be developed in order to help general practitioners to suspect myeloma sooner.

FUNDING

This manuscript presents work carried out as part of a DPhil scholarship awarded to Mr Constantinos Koshiaris funded by the Primary Care Research Trust, the university of Oxford

For peer review only - http://bmjopen.bmj.com/site/about/guidelines.xhtml

and NIHR Oxford CLAHRC. AVDB is supported through the National Institute for Health Research (NIHR) Diagnostic Evidence Co-operative Oxford at Oxford Health Foundation Trust (IS_DEC_0812_100).

The research was supported by the National Institute for Health Research (NIHR) Collaboration for Leadership in Applied Health Research and Care Oxford at Oxford Health NHS Foundation Trust. The views expressed are those of the authors and not necessarily those of the NHS, the NIHR or the Department of Health.

ACKNOWLEDGMENTS

This study has been presented as an oral presentation at the SAPC conference, Dublin July 2016

Six authors contributed to this study: Constantinos Koshiaris MSc, Jason Oke PhD, Lucy Abel MSc, Brian D Nicholson MRCGP, Karthik Ramasamy PhD, and Ann Van den Bruel PhD. CK designed the study with input from AVDB and JO. All authors were involved in the conduct of the study, interpreting the results, and in revising and correcting the manuscript. CK and LA reviewed and extracted the data from articles. CK and JO planned and conducted the analysis. The manuscript drafting was led by CK with the contribution of all authors. All authors read and approved the final version of the manuscript.

CONFLICT OF INTEREST

No authors report a conflict of interest

DATA SHARING STATEMENT

The dataset is available on request from the corresponding author.

For peer review only - http://bmjopen.bmj.com/site/about/guidelines.xhtml

For peer review only

REFERENCES

1 England PH. National Cancer Intelligence Network Cancer statistics : availability and location About Public Health England. 2015.

2 Myeloma statistics | Cancer Research UK. http://www.cancerresearchuk.org/health-professional/cancer-statistics/statistics-by-cancer-type/myeloma#heading-Six (accessed 26 Aug 2016).

3 Quaresma M, Coleman MP, Rachet B. 40-year trends in an index of survival for all cancers combined and survival adjusted for age and sex for each cancer in England and Wales , 1971 – 2011 : a population-based study. *Lancet* 2015;**385**:1206–18. doi:10.1016/S0140-6736(14)61396-9

4 Lyratzopoulos G, Wardle J, Rubin G. Rethinking diagnostic delay in cancer: how difficult is the diagnosis? *Bmj* 2014;**349**:g7400–g7400. doi:10.1136/bmj.g7400

5 Shephard EA, Neal RD, Rose P, *et al.* Quantifying the risk of multiple myeloma from symptoms reported in primary care patients: a large case-control study using electronic records. *Br J Gen Pr* 2015;**65**:e106-13. doi:10.3399/bjgp15X683545

6 Hamilton W, Round A, Sharp D, *et al.* Clinical features of colorectal cancer before diagnosis : a population-based case – control study. 2005;:399–405. doi:10.1038/sj.bjc.6602714

7 Lyratzopoulos G, Neal RD, Barbiere JM, *et al.* Variation in number of general practitioner consultations before hospital referral for cancer: Findings from the 2010 National Cancer Patient Experience Survey in England. *Lancet Oncol* 2012;**13**:353–65. doi:10.1016/S1470-2045(12)70041-4

8 Friese CR, Abel G a., Magazu LS, *et al.* Diagnostic delay and complications for older adults with multiple myeloma. *Leuk Lymphoma* 2009;**50**:392–400. doi:10.1080/10428190902741471

9 Macdonald S, Macleod U, Campbell NC, *et al.* Systematic review of factors influencing patient and practitioner delay in diagnosis of upper gastrointestinal cancer. *Br J Cancer* 2006;**94**:1272–80. doi:10.1038/sj.bjc.6603089

10 Tosi P. Diagnosis and treatment of bone disease in multiple myeloma: spotlight on spinal involvement. *Scientifica (Cairo)* 2013;**2013**:104546. doi:10.1155/2013/104546

11 Sonmez M, Akagun T, Topbas M, *et al.* Effect of pathologic fractures on survival in multiple myeloma patients : a case control study. 2008;**4**:1–4. doi:10.1186/1756-9966-27-Received

12 Wirk B. Renal failure in multiple myeloma: a medical emergency. *Bone Marrow Transplant* 2011;**46**:771–83. doi:10.1038/bmt.2011.8

13 Smith D, Yong K. Multiple myeloma. *Bmj* 2013;**346**:f3863–f3863. doi:10.1136/bmj.f3863

14 Kariyawasan CC, Hughes D a., Jayatillake MM, *et al.* Multiple myeloma: causes and consequences of delay in diagnosis. *Qjm* 2007;**100**:635–40.

For peer review only

doi:10.1093/qjmed/hcm077

15 Ong F, Hermans J, Noordijk EM, *et al.* Presenting signs and symptoms in multiple myeloma: High percentages of stage III among patients without apparent myeloma-associated symptoms. *Ann Hematol* 1995;**70**:149–52. doi:10.1007/s002770050048

16 Greipp PR. International Staging System for Multiple Myeloma. *J Clin Oncol* 2005;**23**:3412–20. doi:10.1200/JCO.2005.04.242

17 Olesen F, Hansen RP, Vedsted P. Delay in diagnosis: the experience in Denmark. *Br J Cancer* 2009;**101 Suppl**:S5–8. doi:10.1038/sj.bjc.6605383

18 Weller D, Vedsted P, Rubin G, *et al.* The Aarhus statement: improving design and reporting of studies on early cancer diagnosis. *Br J Cancer* 2012;**106**:1262–7. doi:10.1038/bjc.2012.68

19 Din NU, Ukoumunne OC, Rubin G, *et al.* Age and Gender Variations in Cancer Diagnostic Intervals in 15 Cancers: Analysis of Data from the UK Clinical Practice Research Datalink. *PLoS One* 2015;**10**:e0127717. doi:10.1371/journal.pone.0127717

20 Neal RD, Din NU, Hamilton W, *et al.* Comparison of cancer diagnostic intervals before and after implementation of NICE guidelines: analysis of data from the UK General Practice Research Database. *Br J Cancer* 2014;**110**:584–92. doi:10.1038/bjc.2013.791

21 Royal College of GPs. National Audit of Cancer Diagnosis in Primary Care. *Networks* 2011.

22 Lyratzopoulos G, Saunders CL, Abel GA, *et al.* The relative length of the patient and the primary care interval in patients with 28 common and rarer cancers. *Br J Cancer* 2015;**112 Suppl**:S35-40. doi:10.1038/bjc.2015.40

23 Lyratzopoulos G, Abel G a, McPhail S, *et al.* Measures of promptness of cancer diagnosis in primary care: secondary analysis of national audit data on patients with 18 common and rarer cancers. *Br J Cancer* 2013;**108**:1–5. doi:10.1038/bjc.2013.1

24 Howell DA, Smith AG, Jack A, *et al.* Time-to-diagnosis and symptoms of myeloma, lymphomas and leukaemias: a report from the Haematological Malignancy Research Network. *BMC Hematol* 2013;**13**:9. doi:10.1186/2052-1839-13-9

25 Varga G, Mikala G, Andrikovics H, *et al.* [How long does a myeloma patient currently wait for the diagnosis in Hungary?]. *Orv Hetil* 2014;**155**:1538–43. doi:10.1556/OH.2014.30000

26 Corner J, Hopkinson J, Fitzsimmons D, *et al.* Is late diagnosis of lung cancer inevitable ? Interview study of patients' recollections of symptoms before diagnosis. 2005;:314–9. doi:10.1136/thx.2004.029264

27 Howell DA, Warburton F, Ramirez A-J, *et al.* Risk factors and time to symptomatic presentation in leukaemia, lymphoma and myeloma. *Br J Cancer* 2015;**113**:1114–20. doi:10.1038/bjc.2015.311

28 Redaniel MT, Martin RM, Ridd MJ, *et al.* Diagnostic intervals and its association with breast, prostate, lung and colorectal cancer survival in England: Historical cohort study

For peer review only - http://bmjopen.bmj.com/site/about/guidelines.xhtml

using the Clinical Practice Research Datalink. *PLoS One* 2015;**10**:1–17. doi:10.1371/journal.pone.0126608

29 Howell D, Smith A, Appleton S, *et al.* Multiple myeloma : routes to diagnosis , clinical characteristics and survival – findings from a UK population-based study. 2017;:1–5. doi:10.1111/bjh.14513

30 Obe EL. Myeloma Patient Experience Report 2016 Foreword. 2016.

31 Rubin GP, Saunders CL, Abel GA, *et al.* Impact of investigations in general practice on timeliness of referral for patients subsequently diagnosed with cancer : analysis of national primary care audit data. *Br J Cancer* 2015;**112**:676–87. doi:10.1038/bjc.2014.634

32 Barrett J, Hamilton W. Pathways to the diagnosis of lung cancer in the UK: a cohort study. *BMC Fam Pract* 2008;**9**:31. doi:10.1186/1471-2296-9-31

33 Jensen H, Tørring ML, Olesen F, *et al.* Cancer suspicion in general practice , urgent referral and time to diagnosis : a population-based GP survey and registry study. 2014.

34 Ades AE, Biswas M, Welton NJ, *et al.* Symptom lead time distribution in lung cancer: Natural history and prospects for early diagnosis. *Int J Epidemiol* 2014;**43**:1865–73. doi:10.1093/ije/dyu174

For peer review only

For peer review only - http://bmjopen.bmj.com/site/about/guidelines.xhtml

Table 1: Study characteristics

Table 1: Study characteristics					
**Myeloma specific**	**Study design**	**Study period**	**Population characteristics (age, gender)**	**Sample size**	**Outcome measure (Interval)**
**Nafees et al 2015, (UK)**					
Multiple cancers	Retrospective analysis (CPRD)	2007-2010	median age 72<br>56% males	500	Diagnostic
**Howell et al 2013, (UK)**					
Haematological cancers	Survey	2004-2011	Median age 69.9<br>66.9% males	341	Patient<br>Diagnostic<br>Total
**Friese et al 2009, (USA)**					
Yes	Retrospective analysis(SEER)	1992-2002	mean age 76.3<br>46% males	3831	Diagnostic
**Lyratzopoulos et al 2013, (UK)**					
Multiple cancers	Audit data	2009-2010	Not reported	176	Primary Care
**Lyratzopoulos et al 2015, (UK)**					
Multiple cancers	Audit data	2009-2010	Not reported	124	Patient
**Varga et al 2014, (Hungary)**					
Yes	Retrospective analysis of medical records	Not reported	median age 60<br>50% males	193	Diagnostic
**Neal et al 2014, (UK)**					
Multiple cancers	Retrospective analysis (CPRD)	2001-2002	mean age 72<br>53% males	221	Diagnostic

For peer review only

For peer review only - http://bmjopen.bmj.com/site/about/guidelines.xhtml

Table 2: Length of intervals

Table 2: Length of intervals			
**Percentile**	**25th**	**50th**	**75th**
**Patient Interval**			
Howell et al 2013	1	31	122
Lyratzopoulos et al 2015	0	13.5	31
Weighted estimate	0.7	26.3	97.7
**Primary care Interval**			
Lyratzopoulos et al 2013	5	21	55
**Secondary care interval**			
No papers reporting secondary care interval			
**Diagnostic interval**			
Nafees et al 2015	54	149	263
Howell et al 2013	34	83	167
Friese et al 2009	27	99	252
Varga et al, 2014	88	125	230
Neal et al, 2014	56	144	264
Weighted estimate	33.7	105.8	247.1
**Total Interval**			
Howell et al, 2013	84	163	306

For peer review only - http://bmjopen.bmj.com/site/about/guidelines.xhtml

Table 3: Symptoms and date definitions

Table 3: Symptoms and interval definitions				
**Symptoms used**	**Onset of first symptom**	**Date of first Presentation in healthcare services**	**Date of first referral**	**Date of diagnosis**
**Nafees et al 2015, (UK)**				
Bleeding<br>Bone Pain<br>Bruising<br>Anaemia<br>Fatigue<br>Anorexia<br>Weight loss	N/A	1 year before diagnosis	N/A	First occurrence of a myeloma Read Code in the patient's record in CPRD database.
**Howell et al 2013, (UK)**				
Tiredness<br>Pain<br>Shortness of breath<br>Infections<br>Joint problems/Fractures<br>Stomach/bowel symptoms<br>Other	Patient reported	Patient reported	N/A	Date provided by the Haematological Malignancy Diagnostic service
**Friese et al 2009, (USA)**				
Anaemia<br>Packed red blood cell transfusion (PRBC)<br>Back pain	N/A	1 year before diagnosis	N/A	SEER cancer diagnosis date
**Lyratzopoulos et al 2013, (UK)**				
Not reported	Estimated based on patient's clinical records.	2 years before diagnosis	Date that the referral letter was sent	Clinical records and hospital correspondence
**Lyratzopoulos et al 2015, (UK)**				
Not reported	N/A	2 years before diagnosis	Date that the referral letter was sent	Clinical records and hospital correspondence
**Varga et al 2014, (Hungary)**				
Bone symptoms<br>Anaemia<br>Renal failure<br>General symptoms<br>Other<br>Tumour presence<br>Metastatic bone disease	N/A	3 years before diagnosis	N/A	Tertiary haematology centre
**Neal et al 2014, (UK)**				
Bleeding<br>Bone Pain<br>Bruising<br>Anaemia<br>Fatigue<br>Anorexia<br>Weight loss	N/A	1 year before diagnosis	N/A	First occurrence of a myeloma Read Code in the patient's record in CPRD database.

For peer review only - http://bmjopen.bmj.com/site/about/guidelines.xhtml

For peer review only

For peer review only - http://bmjopen.bmj.com/site/about/guidelines.xhtml

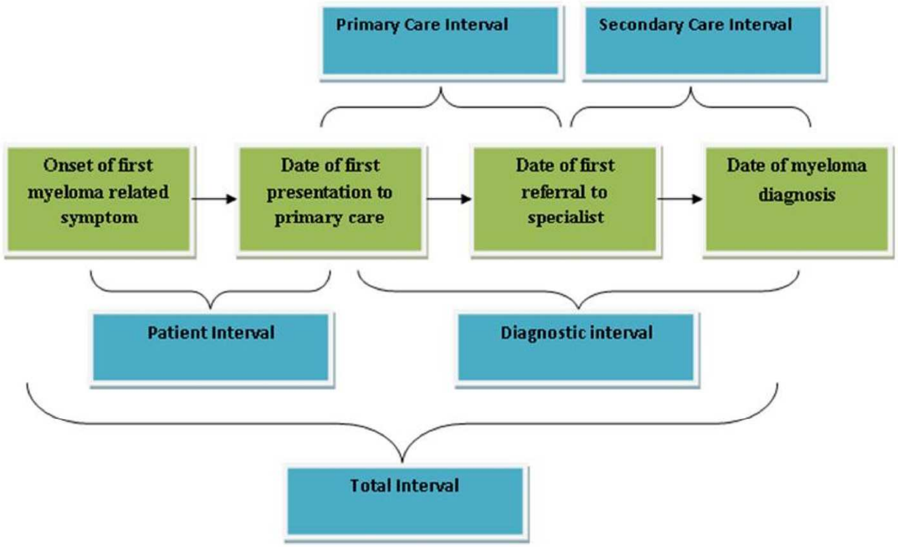

Figure 1 : Outcome definition

157x124mm (300 x 300 DPI)

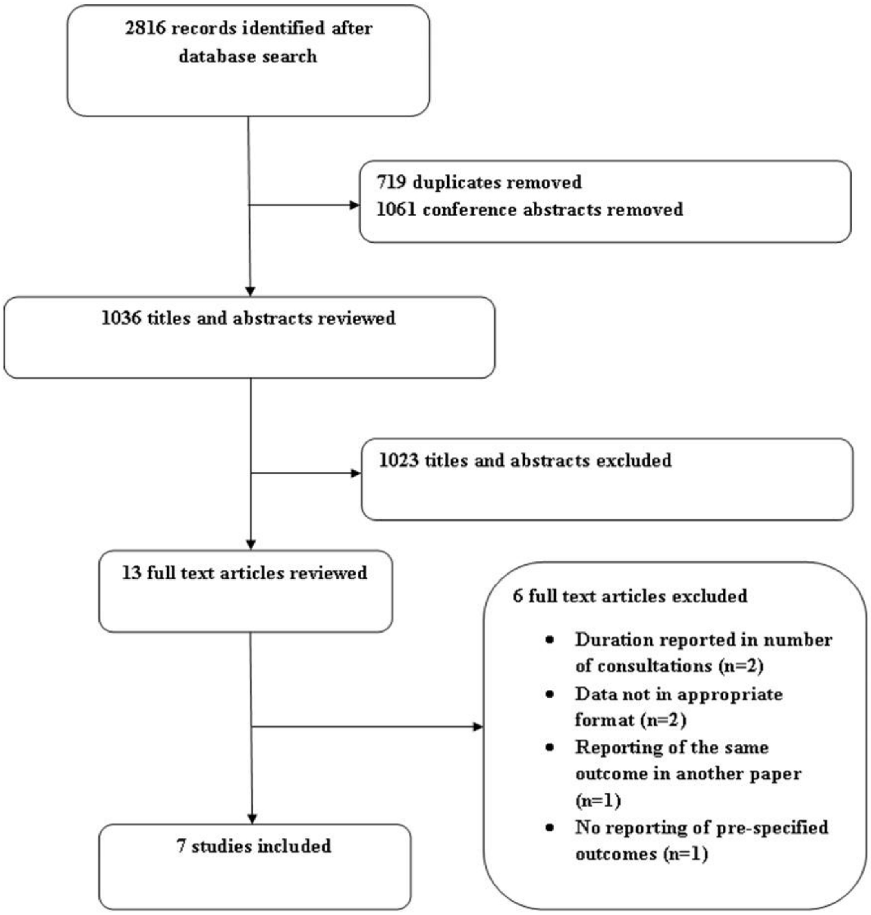

Figure 2: Study selection flowchart

256x331mm (300 x 300 DPI)

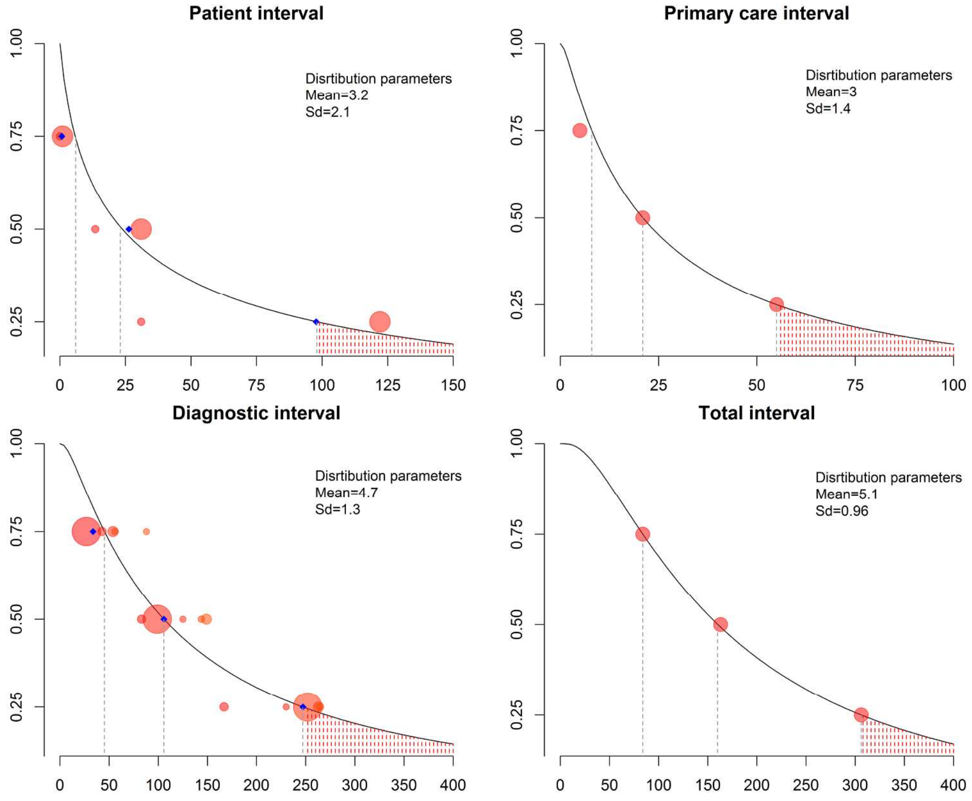

Figure 3: Distribution of intervals. Each circle corresponds to one study and the size is proportional to the total sample size. The blue diamond corresponds to the weighted estimate. For intervals with only one study (primary and total) no weighted estimates were calculated. Y-axis corresponds to 1-Probability (interval > number of days) i.e. 0.25 corresponds to the 75th percentile and 0.75 to the 25th percentile.

237x189mm (300 x 300 DPI)

A1: Search strategy

1. Multiple Myeloma/
2. Myeloma*.ti,ab.
3. 1 or 2
4. (time adj4 diagnos$).ti,ab.
5. (time adj4 consult$).ti,ab.
6. (time adj4 refer$).ti,ab.
7. (time adj4 present$).ti,ab.
8. 4 or 5 or 6 or 7
9. (delay$ adj4 diagnos$).ti,ab.
10. (delay$ adj4 consult$).ti,ab.
11. (delay$ adj4 refer$).ti,ab.
12. (delay$ adj4 present$).ti,ab
13. (delay$adj4 seek$).ti,ab
14. (delay$ adj4 detect*).ti,ab.
15. 9 or 10 or 11 or 12 or 13 or 14
16. (interval adj4 consult$).ti,ab.
17. (interval adj4 refer$).ti,ab.
18. (interval adj4 present$).ti,ab.
19. 16 or 17 or 18
20. (late adj4 diagnosis).ti,ab.
21. (late adj4 detect*).ti,ab.
22. (late adj4 present$).ti,ab.
23. 20 or 21 or 22
24. diagnos$ delay$.ti,ab.
25. early diagnos$.ti,ab.

For peer review only

A2: Risk of bias graph

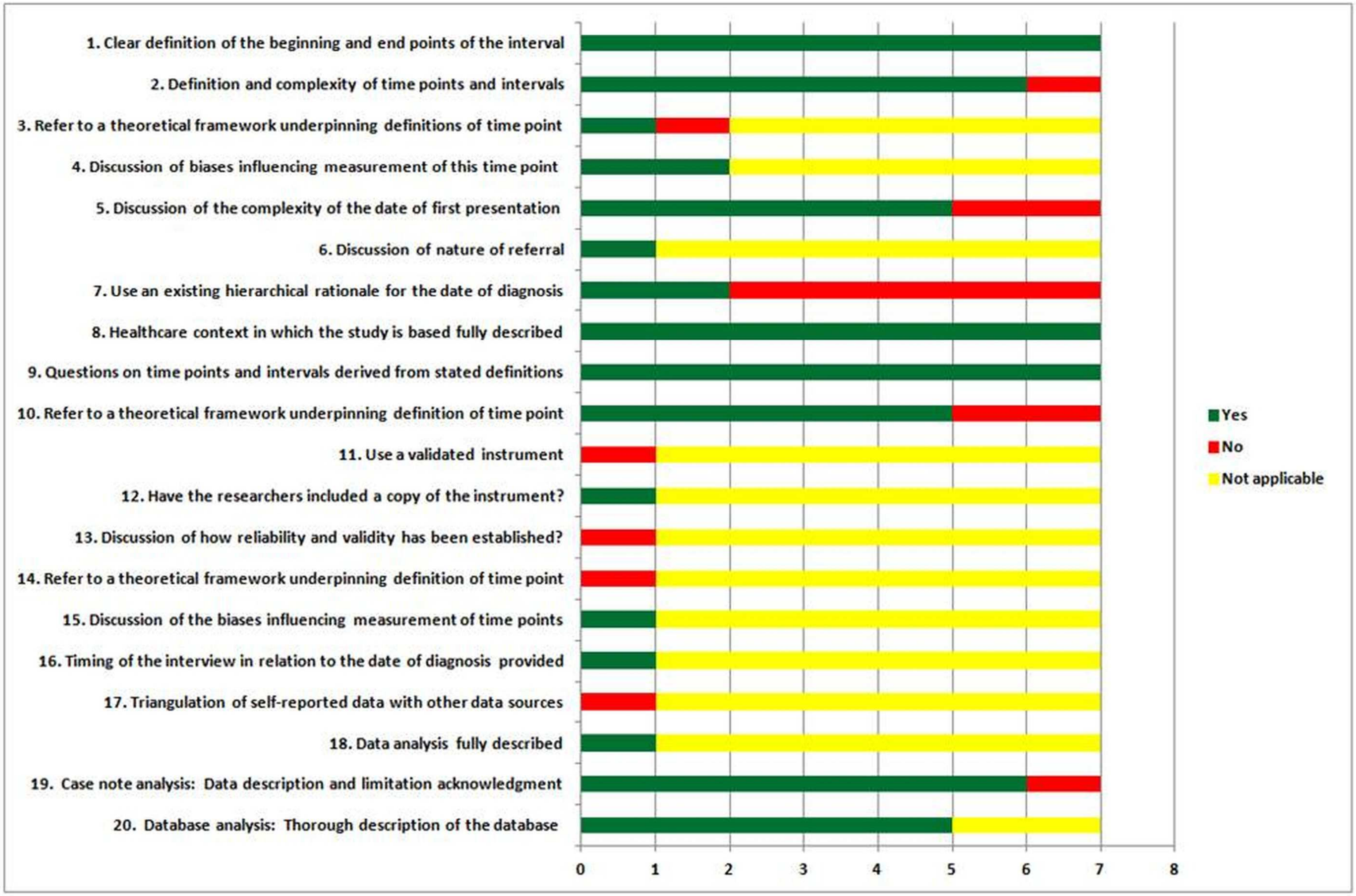

For peer review only - http://bmjopen.bmj.com/site/about/guidelines.xhtml

A3: Risk of bias summary

Legend: No (red) · Yes (green) · Not applicable (yellow)

	1	2	3	4	5	6	7	8	9	10	11	12	13	14	15	16	17	18	19	20
Nafees 2015	Yes	Yes	Not applicable	Not applicable	Yes	Not applicable	Yes	Yes	Yes	Yes	Not applicable	Not applicable	Not applicable	Not applicable	Not applicable	Not applicable	Not applicable	Not applicable	Not applicable	Yes
Howell 2013	Yes	Yes	No	Yes	Yes	Not applicable	No	Yes	Yes	Yes	No	Yes	No	No	Yes	Yes	No	Yes	Not applicable	Not applicable
Friese 2009	Yes	Yes	Not applicable	Not applicable	No	Not applicable	No	Yes	Yes	No	Not applicable	Not applicable	Not applicable	Not applicable	Not applicable	Not applicable	Not applicable	Not applicable	Not applicable	Yes
Lyratzopoulos 2013	Yes	Yes	Not applicable	Not applicable	Yes	Yes	No	Yes	Yes	Yes	Not applicable	Not applicable	Not applicable	Not applicable	Not applicable	Not applicable	Not applicable	Not applicable	Not applicable	Yes
Lyratzopoulos 2015	Yes	Yes	Yes	Yes	Yes	Not applicable	No	Yes	Yes	Yes	Not applicable	Not applicable	Not applicable	Not applicable	Not applicable	Not applicable	Not applicable	Not applicable	Not applicable	Yes
Varga 2014	Yes	No	Not applicable	Not applicable	No	Not applicable	No	Yes	Yes	No	Not applicable	Not applicable	Not applicable	Not applicable	Not applicable	Not applicable	Not applicable	Not applicable	No	Not applicable
Neal 2014	Yes	Yes	Not applicable	Not applicable	Yes	Not applicable	Yes	Yes	Yes	Yes	Not applicable	Not applicable	Not applicable	Not applicable	Not applicable	Not applicable	Not applicable	Not applicable	Not applicable	Yes

For peer review only - http://bmjopen.bmj.com/site/about/guidelines.xhtml

PRISMA 2009 Checklist

Section/topic	#	Checklist item	Reported on page #
**TITLE**			
Title	1	Identify the report as a systematic review, meta-analysis, or both.	1
**ABSTRACT**			
Structured summary	2	Provide a structured summary including, as applicable: background; objectives; data sources; study eligibility criteria, participants, and interventions; study appraisal and synthesis methods; results; limitations; conclusions and implications of key findings; systematic review registration number.	2
**INTRODUCTION**			
Rationale	3	Describe the rationale for the review in the context of what is already known.	4-5
Objectives	4	Provide an explicit statement of questions being addressed with reference to participants, interventions, comparisons, outcomes, and study design (PICOS).	5-6
**METHODS**			
Protocol and registration	5	Indicate if a review protocol exists, if and where it can be accessed (e.g., Web address), and, if available, provide registration information including registration number.	5-6
Eligibility criteria	6	Specify study characteristics (e.g., PICOS, length of follow-up) and report characteristics (e.g., years considered, language, publication status) used as criteria for eligibility, giving rationale.	5-6
Information sources	7	Describe all information sources (e.g., databases with dates of coverage, contact with study authors to identify additional studies) in the search and date last searched.	5-6
Search	8	Present full electronic search strategy for at least one database, including any limits used, such that it could be repeated.	p.5 & Appendix
Study selection	9	State the process for selecting studies (i.e., screening, eligibility, included in systematic review, and, if applicable, included in the meta-analysis).	6
Data collection process	10	Describe method of data extraction from reports (e.g., piloted forms, independently, in duplicate) and any processes for obtaining and confirming data from investigators.	6
Data items	11	List and define all variables for which data were sought (e.g., PICOS, funding sources) and any assumptions and simplifications made.	6
Risk of bias in individual studies	12	Describe methods used for assessing risk of bias of individual studies (including specification of whether this was done at the study or outcome level), and how this information is to be used in any data synthesis.	6
Summary measures	13	State the principal summary measures (e.g., risk ratio, difference in means).	6
Synthesis of results	14	Describe the methods of handling data and combining results of studies, if done, including measures of consistency (e.g., I^2) for each meta-analysis.	6

For peer review only

PRISMA 2009 Checklist

Section/topic	#	Checklist item	Reported on page #
Risk of bias across studies	15	Specify any assessment of risk of bias that may affect the cumulative evidence (e.g., publication bias, selective reporting within studies).	6-7
Additional analyses	16	Describe methods of additional analyses (e.g., sensitivity or subgroup analyses, meta-regression), if done, indicating which were pre-specified.	6-7
**RESULTS**			
Study selection	17	Give numbers of studies screened, assessed for eligibility, and included in the review, with reasons for exclusions at each stage, ideally with a flow diagram.	7 (figure 1)
Study characteristics	18	For each study, present characteristics for which data were extracted (e.g., study size, PICOS, follow-up period) and provide the citations.	7 (table 1)
Risk of bias within studies	19	Present data on risk of bias of each study and, if available, any outcome level assessment (see item 12).	Appendix
Results of individual studies	20	For all outcomes considered (benefits or harms), present, for each study: (a) simple summary data for each intervention group (b) effect estimates and confidence intervals, ideally with a forest plot.	p. 7-9 table 2, figure 3
Synthesis of results	21	Present results of each meta-analysis done, including confidence intervals and measures of consistency.	p. 7-9 table 2, figure 3
Risk of bias across studies	22	Present results of any assessment of risk of bias across studies (see Item 15).	8-9 appendix
Additional analysis	23	Give results of additional analyses, if done (e.g., sensitivity or subgroup analyses, meta-regression [see Item 16]).	9
**DISCUSSION**			
Summary of evidence	24	Summarize the main findings including the strength of evidence for each main outcome; consider their relevance to key groups (e.g., healthcare providers, users, and policy makers).	9-10
Limitations	25	Discuss limitations at study and outcome level (e.g., risk of bias), and at review-level (e.g., incomplete retrieval of identified research, reporting bias).	10-12
Conclusions	26	Provide a general interpretation of the results in the context of other evidence, and implications for future research.	10-13
**FUNDING**			

For peer review only

PRISMA 2009 Checklist

Funding	27	Describe sources of funding for the systematic review and other support (e.g., supply of data); role of funders for the systematic review.	14

From: Moher D, Liberati A, Tetzlaff J, Altman DG, The PRISMA Group (2009). Preferred Reporting Items for Systematic Reviews and Meta-Analyses: The PRISMA Statement. PLoS Med 6(7): e1000097. doi:10.1371/journal.pmed1000097

For more information, visit: www.prisma-statement.org.

For peer review only

MOOSE Checklist for Meta-analyses of Observational Studies

Item No	Recommendation	Reported on Page No
Reporting of background should include		
1	Problem definition	P4
2	Hypothesis statement	P5
3	Description of study outcome(s)	P5
4	Type of exposure or intervention used	NA
5	Type of study designs used	P5
6	Study population	P5
Reporting of search strategy should include		
7	Qualifications of searchers (eg, librarians and investigators)	P5
8	Search strategy, including time period included in the synthesis and key words	P5 & appendix
9	Effort to include all available studies, including contact with authors	P6
10	Databases and registries searched	P5
11	Search software used, name and version, including special features used (eg, explosion)	P5 & appendix
12	Use of hand searching (eg, reference lists of obtained articles)	NA
13	List of citations located and those excluded, including justification	P7/F2
14	Method of addressing articles published in languages other than English	P5
15	Method of handling abstracts and unpublished studies	P5
16	Description of any contact with authors	P6
Reporting of methods should include		
17	Description of relevance or appropriateness of studies assembled for assessing the hypothesis to be tested	P5
18	Rationale for the selection and coding of data (eg, sound clinical principles or convenience)	P5-6
19	Documentation of how data were classified and coded (eg, multiple raters, blinding and interrater reliability)	P5-6
20	Assessment of confounding (eg, comparability of cases and controls in studies where appropriate)	NA
21	Assessment of study quality, including blinding of quality assessors, stratification or regression on possible predictors of study results	P 6-7
22	Assessment of heterogeneity	P 7-8, 10
23	Description of statistical methods (eg, complete description of fixed or random effects models, justification of whether the chosen models account for predictors of study results, dose-response models, or cumulative meta-analysis) in sufficient detail to be replicated	P 7-8
24	Provision of appropriate tables and graphics	T1,T2,T3, F1, F2, F3
Reporting of results should include		
25	Graphic summarizing individual study estimates and overall estimate	T2, F3
26	Table giving descriptive information for each study included	T1
27	Results of sensitivity testing (eg, subgroup analysis)	P9, 11
28	Indication of statistical uncertainty of findings	P10

For peer review only

Item No	Recommendation	Reported on Page No
Reporting of discussion should include		
29	Quantitative assessment of bias (eg, publication bias)	P8
30	Justification for exclusion (eg, exclusion of non-English language citations)	NA
31	Assessment of quality of included studies	P8, appendix
Reporting of conclusions should include		
32	Consideration of alternative explanations for observed results	P10-11
33	Generalization of the conclusions (ie, appropriate for the data presented and within the domain of the literature review)	P11-12
34	Guidelines for future research	P12
35	Disclosure of funding source	P14

From: Stroup DF, Berlin JA, Morton SC, et al, for the Meta-analysis Of Observational Studies in Epidemiology (MOOSE) Group. Meta-analysis of Observational Studies in Epidemiology. A Proposal for Reporting. *JAMA*. 2000;283(15):2008-2012. doi: 10.1001/jama.283.15.2008.

Transcribed from the original paper within the NEUROSURGERY® Editorial Office, Atlanta, GA, United Sates. August 2012.

For peer review only

BMJ Open

Quantifying intervals to diagnosis in myeloma: a systematic review and meta-analysis

Journal:	*BMJ Open*
Manuscript ID	bmjopen-2017-019758.R1
Article Type:	Research
Date Submitted by the Author:	13-Feb-2018
Complete List of Authors:	Koshiaris, Constantinos; University of Oxford Department of Primary Care Health Sciences<br>Oke, Jason; University of Oxford, Nuffiled Department of Primary Care Health Sciences<br>Abel, Lucy; University of Oxford, Nuffield Department of Primary Care Health Sciences<br>Nicholson, Brian; University of Oxford, Nuffield Dept Primary Care Health Sciences<br>Ramasamy, Karthik ; Churchill Hospital, Department of Haematology, Oxford University Hospitals NHS Trust<br>van den Bruel, Ann; University of Oxford, Dept of Primary Health Care
**Primary Subject Heading**:	Haematology (incl blood transfusion)
Secondary Subject Heading:	Health services research
Keywords:	Myeloma < HAEMATOLOGY, time to diagnosis, early diagnosis, PRIMARY CARE, systematic review, delays

SCHOLARONE™
Manuscripts

Quantifying intervals to diagnosis in myeloma: a systematic review and meta-analysis

Constantinos Koshiaris[1] MSc, Jason Oke[1] PhD, Lucy Abel[1] MSc, Brian D Nicholson[1] MRCGP, Karthik Ramasamy[2 3] PHD, Ann Van den Bruel PHD[1]

[1]Nuffield Department of Primary Care Health Sciences, University of Oxford, Radcliffe Primary Care Building, Radcliffe Observatory Quarter, Woodstock Road, Oxford OX2 6GG

[2] Department of Haematology, Oxford University Hospitals NHS Trust, Old Road, Headington, Oxford OX3 7LE

[3] NIHR BRC Blood Theme, Oxford

Corresponding author:

Mr. Constantinos Koshiaris (constantinos.koshiaris@phc.ox.ac.uk)

Telephone number: 01865 289317

Nuffield Department of Primary Care Health Sciences, University of Oxford, Radcliffe Primary Care Building, Radcliffe Observatory Quarter, Woodstock Road, Oxford OX2 6GG

Word count: 3510

Key words: multiple myeloma, time to diagnosis, early diagnosis, primary care, systematic review, delays

For peer review only

ABSTRACT

Objectives

To quantify the duration of each step of the diagnostic pathway for multiple myeloma patients from symptom onset to confirmation of diagnosis

Design

Systematic review and meta-analysis

Data sources and selection criteria

The MEDLINE and EMBASE databases were searched up until January 2018 to identify articles which reported time intervals from onset of symptoms to diagnosis. Articles focusing on children or adolescents and on the asymptomatic form of the disease (monoclonal gammopathies and smouldering myeloma) were excluded.

Data collection and data analysis

Data were extracted independently by two reviewers. Weighted estimates of the median and interquartile range were calculated. Risk of bias was assessed using the Aarhus checklist.

Main results

Nine studies were included. The patient interval (first symptom – first presentation) has a median of 26.3 days (IQR: 1 to 98, n=465, 2 studies). Subsequently, the primary care (first presentation – first referral) interval is 21.6 days (IQR: 4.6 to 55.8, n=326, 2 studies), the diagnostic interval (first presentation – diagnosis) 108.6 days (IQR: 33.3 to 241.7, n=5395, 7 studies) and the time to diagnosis (first symptom – diagnosis) interval 163 days (IQR: 84 to 306, n=341, 1 study). No studies were describing the referral to diagnosis interval.

Conclusion

The review demonstrates that there is scope for significant reductions in the time to myeloma diagnosis. At present, many patients experience a diagnostic interval longer than 3 months until diagnosis is confirmed.

For peer review only - http://bmjopen.bmj.com/site/about/guidelines.xhtml

Review registration

Not available. Protocol available from authors

Strengths and limitations of this study

Strengths

- First systematic review that quantified the whole diagnostic pathway for multiple myeloma patients including the different intervals in each step of the pathway
- Use of all available information including the interquartile range rather than just focusing on measures of central tendency like the mean and the median

Limitations

- No universally accepted methods for formal meta-analysis of median and interquartile range
- Limited number of studies reporting the patient and primary care intervals and no studies were reporting the referral to diagnosis interval so any inferences regarding the referral to diagnosis interval should be interpreted with caution.

For peer review only

For peer review only - http://bmjopen.bmj.com/site/about/guidelines.xhtml

INTRODUCTION

Myeloma is a haematological malignancy characterised by uncontrolled plasma cell production in the bone marrow. It was the 17th most common cancer in the UK in 2013 accounting for 2% of all new cancer cases. Currently there are more than 17500 myeloma patients in the UK with approximately 5500 cases being diagnosed every year[1,2]. It is a cancer that mainly affects the elderly population with 59% of the patients being diagnosed over the age of 70[2] and with a 5 year survival of 47%[3].

It is considered one of the hardest cancers to suspect in primary care. Symptoms of myeloma are very common in other conditions as well, such as back pain, bone pain, fatigue and repeated infections[4]. This in combination with the fact that myeloma is a very rare condition in primary care results in very low predictive values for individual symptoms. For example, primary care patients with back pain, which is one of the most common myeloma symptoms, only have a 0.1% risk of myeloma[5]. By comparison, patients with rectal bleeding have a 2.4% risk of colorectal cancer[6].

As a result, half of symptomatic myeloma patients have three or more consultations in primary care before they are referred to specialist care which is more than in any other cancer[7]. Attributing symptoms to comorbidities further prolongs the diagnostic process, which is particularly relevant in this older age group [8,9].

Delays in diagnosing myeloma allow complications to develop (end organ damage), such as pathological fractures, irreversible renal failure and in some cases spinal cord compression[10–12]. These are considered medical emergencies in their own right and limit the opportunity for applying effective treatment[13]. A delayed diagnosis is also linked with higher cancer stage[14,15] which is in turn associated with poorer survival[16]. Patients with

For peer review only

For peer review only - http://bmjopen.bmj.com/site/about/guidelines.xhtml

longer diagnostic intervals also experience shorter disease free survival and more complications from treatment[14].

Quantifying the time-intervals leading up to diagnosis is important as it will inform future interventions that aim to shorten the diagnostic process. The aim of this systematic review was to quantify each step of the diagnostic pathway to myeloma diagnosis and identify where to focus efforts to reduce diagnostic delay.

METHODS

A protocol is available on request from the authors. A copy of the search strategy can be seen in the appendix (A1). We searched EMBASE and MEDLINE until January 2018 for studies that quantified any or all of the following five intervals[17]: the patient interval (from symptom onset to first consultation); the primary care interval (from first consultation for that symptom to referral to secondary care); the diagnostic interval (from first consultation with a myeloma related symptom to diagnosis) and the time to diagnosis (from symptom onset to diagnosis). In addition we looked for studies that were estimating the Referral to diagnosis interval.(Figure 1). Citation searching of key references like the Aarhus statement was conducted and we also searched the reference list of systematic reviews with similar research questions [18,19]. We included any study designs that quantified at least one of the intervals mentioned above in days or months. Studies reporting the length of an interval only in number of consultations or referrals were excluded as were studies focusing on children or adolescents (< 18 years) and on the asymptomatic forms of the disease (monoclonal gammopathies and smouldering myeloma). We included only papers with an abstract in English, but did not exclude full text articles based on language (as long as there was an English abstract). Conference abstracts were excluded. Two reviewers (CK/LA) selected papers for inclusion using the criteria listed above, on title and abstracts first and on full text second. Disagreements were resolved through discussion with a third reviewer (JO/AVB).

For peer review only

Data extraction

Two reviewers (CK/LA) independently extracted data from the included studies into a pre-defined spreadsheet. Study characteristics including author, year of publication, country of data collection, type of study, myeloma related symptoms and sample size were extracted, as well as descriptive statistics including median, interquartile range, range, mean and the standard deviation (SD) for each interval. Authors were contacted if data were not available or not in the appropriate format for extraction (i.e. categorical rather than continuous).

Risk of bias assessment

The risk of bias was assessed by two independent researchers (CK/LA) using the Aarhus checklist[18]. The Aarhus checklist is a 20 item tool designed to help researchers design and evaluate studies on early diagnosis of cancer and examines studies in terms of acknowledgment of the different biases influencing time point measurement and interval definition, questionnaire validation and data collection in patient reported data and analysis of case-note audits and databases. We assessed clinical heterogeneity of the included studies in terms of time-points, interval and symptom definitions.

Analysis

In the context of illness duration, intervals are usually not normally distributed, therefore we used the median and interquartile-range (IQR) to summarise the data. We present the 25th, 50th and 75th percentiles for all intervals. When more than one studies were available, we pooled the results by calculating a weighted mean for each percentile where the weight was obtained by dividing the sample size in each study with the total numbers of patients. We also fitted a distribution through the 3 weighted percentile estimates where appropriate in order to try and generate the shape of the distribution of the interval under investigation. We chose the

For peer review only - http://bmjopen.bmj.com/site/about/guidelines.xhtml

For peer review only

lognormal distribution as time intervals are usually skewed to the right[4]. A pre-specified sensitivity analysis was conducted by excluding the study with the higher risk of bias. The sensitivity analysis was conducted only for the diagnostic interval as the rest of the outcomes were reported by only 1 or 2 papers.

RESULTS

We identified 3343 citations from the EMBASE and MEDLINE searches. After removal of conference abstracts and duplicates, we screened 1271 titles and abstracts and 16 studies were candidate for inclusion. Nine studies were included in the final analysis (Figure 2). Seven papers in total were excluded for the following reasons: two because they were reporting the duration in numbers of consultations [5,7]; one because none of the pre-specified outcomes were reported[20]; one reported the same outcome based on the same database as one of the other included papers so inclusion of this paper in the data synthesis would result in double counting [21]; two papers because data were not in an appropriate format [14,22]; one because the interval under investigation was reported only for patients that were referred to very specific specialisations making it a very selective population compared to the other studies [23]

Study Characteristics

A summary of all the included papers is provided in table 1. Studies were published between 2009 and 2018 and the sample size ranged from 107 to 3831 patients. Five studies reported intervals in various cancers[24–28] ; three reported only myeloma[8,29,30]; and one for haematological malignancies[31].

Six studies were conducted in the UK[24–28,31], one in the USA[8], one in Hungary[29] and one in Israel[30]. Two UK studies used data from two separate Clinical Practice Research Datalink (CPRD) cohorts[24,25], a database of routinely collected electronic primary care

For peer review only - http://bmjopen.bmj.com/site/about/guidelines.xhtml

For peer review only

records. Two other UK studies used data from the English National Audit of Cancer Diagnosis in Primary Care 2009-2010[32]: we extracted primary care interval data from the larger study and the patient interval from the smaller study[26,27]Another UK study used data from the English National Audit of Cancer Diagnosis in Primary care 2014 and the last UK study was a patient survey on patients diagnosed with haematological malignancies[28,31]. The study conducted in Hungary analysed data collected from patients treated in a haematology centre and the study conducted in the USA analysed a retrospective database collected from the Surveillance, Epidemiology, and End Results Program (SEER) program[8,29]. The study conducted in Israel used data from the Israeli health maintenance (HMO) organisation linked with the Israel National Cancer Registry (NCR) [30].

Definition of intervals to diagnosis

There was substantial heterogeneity in the symptoms and time points used to define each interval (table 2). In total, 19 different symptoms were used to define the start of myeloma but studies varied greatly regarding which symptom (or symptoms) was used, ranging from 3 to a maximum of 12 symptoms. Three studies did not report the starting symptoms [26–28]. Also some studies included multiple symptoms in more general categories[24,25,31]. For example, Howell et al used a general pain category which included musculoskeletal, abdominal, chest and other type of pains while the two CPRD studies included multiple musculoskeletal symptoms under a general bone pain category[24,25].

The start of the measuring period was defined as the date of onset of the first symptom or the date of first presentation for a myeloma related symptom depending on whether the studies were investigating the patient interval, the diagnostic or both. Out of nine studies, three identified the first symptom within the year preceding diagnosis, one at three years, three at two years before diagnosis and one study used patient reported dates. Goldschmidt N et al

For peer review only

[30] did not use the first symptom as the start of the measurement period but they used the first combination of symptom and laboratory result (i.e. the earliest of blood test + pain complaint or two blood tests within a month or two pain complaints within 1-3 months).

Risk of Bias

Most of the studies included in the analysis had a low risk of bias (Appendix A2, A3). All studies clearly defined the start and end point of the intervals and in most cases there was an adequate description of the databases along with the strengths, limitations and biases arising from the definitions of the different intervals and time point. Only one study did not mention the different limitations and biases arising from the study design and the choice of definitions for time points and intervals[29]. Most common sources of bias that were described included recall bias for studies that were using patient reported data and misclassification bias for studies that were using databases like CPRD. Most studies used a theoretical framework to define each interval usually the one reported by Olesen et al[17] or the Aarhus statement[18]. The category with the higher risk of bias was the use of a hierarchical rationale to determine the date of diagnosis i.e. date of first histological confirmation of the malignancy or date of admission to the hospital for example. Most studies mentioned how the date of diagnosis was obtained but there was no adequate description on how the choice of a particular definition can affect the diagnostic pathway.

Quantifying intervals

Seven papers reported the diagnostic interval [8,24,25,28–31], two the patient interval[27,31], two the primary care interval[26,28], and one the time to diagnosis interval[31]. No studies reported the referral to diagnosis interval. The length of the different intervals can be seen in table 3 and the fitted log normal distributions in figure 3 along with the parameters used to fit them.

For peer review only - http://bmjopen.bmj.com/site/about/guidelines.xhtml

For peer review only

For the diagnostic interval, the pooled weighted mean of the 50th percentile is 108.6 days (n=5398) and the IQR is from 33.3 to 241.7 (n=5288). Removing the study with the largest risk of bias[29] based on the Aarhus statement checklist did not alter the results (107.9 days, IQR: 31.3-242.2). An additional sensitivity analysis was conducted by excluding the Goldschmidt N et al [30] study as it was an outlier but the results were not affected (pooled median of 103.8). While all the studies reported a median diagnostic interval less than 5 months this study had an interval of 11.2 months. The IQR was not estimated for this sensitivity analysis as it was not reported by the authors.

The pooled estimate of the 50th percentile of the patient interval is 26.3 days (IQR: 0.7-97.7, n=465). The primary care interval was reported by two studies [26,28] with a median of 21.6 days (IQR: 4.6-55.8, n=326) and the time to diagnosis interval was also reported by one study[31] with a median of 163 days (IQR: 84-306, n=341).

No study reported the referral to diagnosis interval but it can be inferred by subtracting the median length of the primary care and patient intervals from the diagnostic interval or the time to diagnosis interval which suggests that the median length of the referral to diagnosis interval can range from 60.7 to 115.1 days depending on whether we use the diagnostic or the time to diagnosis interval for the inference.

DISCUSSION

Our results show that myeloma patients experience symptoms for a median of approximately one month before seeking help and 25% of patients wait for more than three months (98 days). After attending primary care with symptoms, the median time to diagnosis is 108.6 days (IQR: 33.3-241.7) with 25% of patients waiting longer than eight months. Inference suggests that the referral to diagnosis interval might be longer than the primary care interval.

Strengths and weaknesses

For peer review only - http://bmjopen.bmj.com/site/about/guidelines.xhtml

This is the first systematic review that quantified the patient pathway of myeloma from onset of first symptom to diagnosis. There were no restrictions in the search strategy in terms of study design or health care systems. We focused our search on two medical databases that are more likely to contain papers on diagnostic pathways but we acknowledge that this might have affected the identification of all literature. To counter this, we included additional strategies like citation searching of some key references and searching the reference lists of similar reviews.

We excluded conference abstracts, although there were several that addressed the review question. The reason for this is that the length of the different intervals reported is affected by design decisions like the choice of when to start the measurement period, initial symptoms, data collection methods etc. Conference abstracts do not report this level of detail in their methods, and therefore could not be included.

In addition to measures of central tendency like the median we included the 25th and 75th percentiles, which are particularly important since time interval data are skewed to the right. Examining all three can provide a more complete idea of the delays that the patients experience especially at the tails of the distribution. Measures of central tendency like the mean might not be the most appropriate to describe the distribution as they tend to be overestimated when used on positively skewed distributions.

The main limitation of the analysis is that currently there are no universally accepted formal methods to perform meta-analysis of medians and interquartile ranges. To overcome this, we combined estimates of the percentiles after weighting them based on the sample size: a method equivalent to a fixed effects meta-analysis. Our estimates of the diagnostic interval might therefore be an underestimation, due to the fact that the biggest study reported one the lowest diagnostic intervals [8]. This however does not change the interpretation of the results

For peer review only - http://bmjopen.bmj.com/site/about/guidelines.xhtml

as these estimates still suggest very long diagnostic intervals for myeloma patients. We were not able to produce a confidence interval around the median and interquartile range as these are not usually measures that are reported by the included studies, thus we present only the point estimate of each percentile. Also we were not able to estimate measures of statistical heterogeneity, although we suspect it to be high.

No studies reported the referral to diagnosis interval and it was inferred based on the results of the other interval so our results regarding this interval should be interpreted with caution.

Sources of heterogeneity

In order to compute intervals, the definition of the beginning and the end of the interval is crucial. There was variability in how studies defined starting points, especially for the first symptom and the first presentation to healthcare, using medical records or patient recall. Studies that use patient reported outcomes tend to suffer from recall bias which might lead to overestimation or underestimation of the different intervals while studies using medical records tend to suffer from loss to follow up and misclassification.

For studies that were using electronic health records there was no agreement on when exactly myeloma starts to manifest. The time used to detect related symptoms prior to diagnosis spanned from one to three years. Although most studies used one year before diagnosis, there is some evidence to suggest that symptoms might be present for more than one year[33] which may have led to an underestimation of intervals in these studies. On the other hand, the more you extend the symptom period the more likely you are to detect symptoms that are unrelated to myeloma which leads to the overestimation of the length of the intervals, especially with symptoms that are so aspecific such as back pain. In order to explore this we conducted a sensitivity analysis where we estimated the length of the diagnostic interval by stratifying according to the time used to define the presenting

For peer review only - http://bmjopen.bmj.com/site/about/guidelines.xhtml

symptoms with studies going one year back having a median of 105 days vs. more than one year back having a median of 142.3 day which might be explaining some of the observed variability.

Findings compared to existing research

Our estimate for the patient interval is in-line with the findings of another study[22] which reported that 15% of myeloma patients wait more than three months before they go to the doctor. This study explained this delay in terms of patients' lack of understanding of the seriousness of their symptoms because of their non-specific nature.

The diagnostic interval, which takes place in health care and could potentially be amenable to improvement, is longer for myeloma than for many other cancers. It has been shown that only 17.2% of myeloma patients are referred through the suspected cancer referral pathway ("two-week" wait) which is lower than other cancers like breast for example which is approximately 43% [20,34]. This could be due to the non-specific nature of the symptoms which make it hard for both the GP and the patient to suspect the presence of myeloma. This might also explain the difference in the length of the diagnostic intervals between these two cancers as the median diagnostic interval for breast cancer is approximately 14 days [35].Other cancers with a similarly long median diagnostic interval also have non-specific clinical presentations, such as lung cancer which has a diagnostic interval of 88 days[35] and leukaemia (all types of leukaemia including chronic and acute) with a median of 102 days[24].

Implications for clinical practice

Trying to reduce intervals matters because longer intervals may be associated with more advanced disease[34]. In addition it has also been reported by the Myeloma Patient

For peer review only - http://bmjopen.bmj.com/site/about/guidelines.xhtml

Experience Report 2016 that more myeloma patients felt their health got worse while waiting to see a specialist than other cancer patients[36].

The primary care interval may be influenced by time spent waiting for the results of further investigations prior to referral[37]. Although investigations such as blood tests may prolong the primary care interval, patients may still benefit if they result in better targeted referrals, reducing the referral to diagnosis interval by avoiding an inappropriate referral which potentially can take more time than the time it takes for a blood result to come back although this is not something that we can say with certainty given our data.

Implications for further research

Although we were not able to estimate the referral to diagnosis care interval directly, it is reasonable to believe that it is longer than the primary care interval but more research is required to validate this claim. Swann et al.[28] estimated the interval from referral to the date the patients were informed that they had cancer to have a median of 35 days for myeloma patients which is longer than the estimate of the primary care interval event thought the definitions of the intervals used in that study and our review are different. As in other cancers[38], referrals to different specialties or with an insufficient level of urgency, or multiple referrals can prolong the referral to diagnosis interval. The choice of referral route has been shown to be a strong predictor of the length of the diagnostic interval[39]. Future studies should not only estimate the duration of primary and referral to diagnosis intervals, but also investigate the impact of one setting on the other as in most cases the type and severity of symptoms will determine the speciality and urgency of referral.

Also it is still not clear how long before diagnosis myeloma symptoms start to occur. In lung cancer, studies on symptom lead time (the time between symptoms attributable to cancer and

For peer review only - http://bmjopen.bmj.com/site/about/guidelines.xhtml

diagnosis) show that symptom incidence increases considerably 6 months before diagnosis[40] but no such study has been conducted for myeloma.

CONCLUSION

Our work suggests that there is potential for meaningful reductions in the time to diagnosis which could improve patient outcomes. Although the time from referral to secondary care to diagnosis appears to be the longest, we believe that shortening the length of this interval could also be in the hands of the general practitioner as they choose the speciality and urgency of referral. More research is required to examine the myeloma patient pathway in more detail, including a more detailed breakdown of referral patterns in myeloma patients in terms of speciality and urgency. More decision making tools should be developed in order to help general practitioners to suspect myeloma sooner.

FUNDING

This manuscript presents work carried out as part of a DPhil scholarship awarded to Mr Constantinos Koshiaris funded by the Primary Care Research Trust, the university of Oxford and NIHR Oxford CLAHRC. AVDB is supported through the National Institute for Health Research (NIHR) Diagnostic Evidence Co-operative Oxford at Oxford Health Foundation Trust (IS_DEC_0812_100).

The research was supported by the National Institute for Health Research (NIHR) Collaboration for Leadership in Applied Health Research and Care Oxford at Oxford Health NHS Foundation Trust. The views expressed are those of the authors and not necessarily those of the NHS, the NIHR or the Department of Health.

ACKNOWLEDGMENTS

For peer review only - http://bmjopen.bmj.com/site/about/guidelines.xhtml

This study has been presented as an oral presentation at the SAPC conference, Dublin July 2016

Six authors contributed to this study: Constantinos Koshiaris MSc, Jason Oke PhD, Lucy Abel MSc, Brian D Nicholson MRCGP, Karthik Ramasamy PhD, and Ann Van den Bruel PhD. CK designed the study with input from AVDB and JO. All authors were involved in the conduct of the study, interpreting the results, and in revising and correcting the manuscript. CK and LA reviewed and extracted the data from articles. CK and JO planned and conducted the analysis. The manuscript drafting was led by CK with the contribution of all authors. All authors read and approved the final version of the manuscript.

CONFLICT OF INTEREST

No authors report a conflict of interest

DATA SHARING STATEMENT

The dataset is available on request from the corresponding author.

For peer review only

REFERENCES

1 England PH. National Cancer Intelligence Network Cancer statistics : availability and location About Public Health England. 2015.

2 Myeloma statistics | Cancer Research UK. http://www.cancerresearchuk.org/health-professional/cancer-statistics/statistics-by-cancer-type/myeloma#heading-Six (accessed 26 Aug 2016).

3 Quaresma M, Coleman MP, Rachet B. 40-year trends in an index of survival for all cancers combined and survival adjusted for age and sex for each cancer in England and Wales , 1971 – 2011 : a population-based study. *Lancet* 2015;**385**:1206–18. doi:10.1016/S0140-6736(14)61396-9

4 Lyratzopoulos G, Wardle J, Rubin G. Rethinking diagnostic delay in cancer: how difficult is the diagnosis? *Bmj* 2014;**349**:g7400–g7400. doi:10.1136/bmj.g7400

5 Shephard EA, Neal RD, Rose P, *et al.* Quantifying the risk of multiple myeloma from symptoms reported in primary care patients: a large case-control study using electronic records. *Br J Gen Pr* 2015;**65**:e106-13. doi:10.3399/bjgp15X683545

6 Hamilton W, Round A, Sharp D, *et al.* Clinical features of colorectal cancer before diagnosis : a population-based case – control study. 2005;:399–405. doi:10.1038/sj.bjc.6602714

7 Lyratzopoulos G, Neal RD, Barbiere JM, *et al.* Variation in number of general practitioner consultations before hospital referral for cancer: Findings from the 2010 National Cancer Patient Experience Survey in England. *Lancet Oncol* 2012;**13**:353–65. doi:10.1016/S1470-2045(12)70041-4

8 Friese CR, Abel G a., Magazu LS, *et al.* Diagnostic delay and complications for older adults with multiple myeloma. *Leuk Lymphoma* 2009;**50**:392–400. doi:10.1080/10428190902741471

9 Macdonald S, Macleod U, Campbell NC, *et al.* Systematic review of factors influencing patient and practitioner delay in diagnosis of upper gastrointestinal cancer. *Br J Cancer* 2006;**94**:1272–80. doi:10.1038/sj.bjc.6603089

10 Tosi P. Diagnosis and treatment of bone disease in multiple myeloma: spotlight on spinal involvement. *Scientifica (Cairo)* 2013;**2013**:104546. doi:10.1155/2013/104546

11 Sonmez M, Akagun T, Topbas M, *et al.* Effect of pathologic fractures on survival in multiple myeloma patients : a case control study. 2008;**4**:1–4. doi:10.1186/1756-9966-27-Received

12 Wirk B. Renal failure in multiple myeloma: a medical emergency. *Bone Marrow Transplant* 2011;**46**:771–83. doi:10.1038/bmt.2011.8

13 Smith D, Yong K. Multiple myeloma. *Bmj* 2013;**346**:f3863–f3863. doi:10.1136/bmj.f3863

14 Kariyawasan CC, Hughes D a., Jayatillake MM, *et al.* Multiple myeloma: causes and consequences of delay in diagnosis. *Qjm* 2007;**100**:635–40.

For peer review only - http://bmjopen.bmj.com/site/about/guidelines.xhtml

doi:10.1093/qjmed/hcm077

15 Ong F, Hermans J, Noordijk EM, *et al.* Presenting signs and symptoms in multiple myeloma: High percentages of stage III among patients without apparent myeloma-associated symptoms. *Ann Hematol* 1995;**70**:149–52. doi:10.1007/s002770050048

16 Greipp PR. International Staging System for Multiple Myeloma. *J Clin Oncol* 2005;**23**:3412–20. doi:10.1200/JCO.2005.04.242

17 Olesen F, Hansen RP, Vedsted P. Delay in diagnosis: the experience in Denmark. *Br J Cancer* 2009;**101 Suppl**:S5–8. doi:10.1038/sj.bjc.6605383

18 Weller D, Vedsted P, Rubin G, *et al.* The Aarhus statement: improving design and reporting of studies on early cancer diagnosis. *Br J Cancer* 2012;**106**:1262–7. doi:10.1038/bjc.2012.68

19 Neal RD, Tharmanathan P, France B, *et al.* Is increased time to diagnosis and treatment in symptomatic cancer associated with poorer outcomes? Systematic review. *Br J Cancer* 2015;**112 Suppl**:S92-107. doi:10.1038/bjc.2015.48

20 Elliss-Brookes L, McPhail S, Ives A, *et al.* Routes to diagnosis for cancer - determining the patient journey using multiple routine data sets. *Br J Cancer* 2012;**107**:1220–6. doi:10.1038/bjc.2012.408

21 Keeble S, Abel GA, Saunders CL, *et al.* Variation in promptness of presentation among 10,297 patients subsequently diagnosed with one of 18 cancers: Evidence from a National Audit of Cancer Diagnosis in Primary Care. *Int J Cancer* 2014;**135**:1220–8. doi:10.1002/ijc.28763

22 Howell DA, Warburton F, Ramirez A-J, *et al.* Risk factors and time to symptomatic presentation in leukaemia, lymphoma and myeloma. *Br J Cancer* 2015;**113**:1114–20. doi:10.1038/bjc.2015.311

23 Li N, Lv Y, Zeng H, *et al.* Renal impairment in multiple myeloma: Presenting features in different departments. *J Huazhong Univ Sci Technol [Medical Sci* 2012;**32**:65–8. doi:10.1007/s11596-012-0011-0

24 Din NU, Ukoumunne OC, Rubin G, *et al.* Age and Gender Variations in Cancer Diagnostic Intervals in 15 Cancers: Analysis of Data from the UK Clinical Practice Research Datalink. *PLoS One* 2015;**10**:e0127717. doi:10.1371/journal.pone.0127717

25 Neal RD, Din NU, Hamilton W, *et al.* Comparison of cancer diagnostic intervals before and after implementation of NICE guidelines: analysis of data from the UK General Practice Research Database. *Br J Cancer* 2014;**110**:584–92. doi:10.1038/bjc.2013.791

26 Lyratzopoulos G, Abel G a, McPhail S, *et al.* Measures of promptness of cancer diagnosis in primary care: secondary analysis of national audit data on patients with 18 common and rarer cancers. *Br J Cancer* 2013;**108**:1–5. doi:10.1038/bjc.2013.1

27 Lyratzopoulos G, Saunders CL, Abel GA, *et al.* The relative length of the patient and the primary care interval in patients with 28 common and rarer cancers. *Br J Cancer* 2015;**112 Suppl**:S35-40. doi:10.1038/bjc.2015.40

For peer review only - http://bmjopen.bmj.com/site/about/guidelines.xhtml

28 Swann R, McPhail S, Witt J, *et al.* Diagnosing cancer in primary care: results from the National Cancer Diagnosis Audit. *Br J Gen Pract* 2018;**68**:e63–72. doi:10.3399/bjgp17X694169

29 Varga G, Mikala G, Andrikovics H, *et al.* [How long does a myeloma patient currently wait for the diagnosis in Hungary?]. *Orv Hetil* 2014;**155**:1538–43. doi:10.1556/OH.2014.30000

30 Goldschmidt N, Zamir L, Poperno A, *et al.* Presenting Signs of Multiple Myeloma and the Effect of Diagnostic Delay on the Prognosis. *J Am Board Fam Med* 2016;**29**:702–9. doi:10.3122/jabfm.2016.06.150393

31 Howell DA, Smith AG, Jack A, *et al.* Time-to-diagnosis and symptoms of myeloma, lymphomas and leukaemias: a report from the Haematological Malignancy Research Network. *BMC Hematol* 2013;**13**:9. doi:10.1186/2052-1839-13-9

32 RCGP, Royal College of GPs. National Audit of Cancer Diagnosis in Primary Care. *Networks* 2011.

33 Corner J, Hopkinson J, Fitzsimmons D, *et al.* Is late diagnosis of lung cancer inevitable ? Interview study of patients' recollections of symptoms before diagnosis. 2005;:314–9. doi:10.1136/thx.2004.029264

34 Howell D, Smith A, Appleton S, *et al.* Multiple myeloma : routes to diagnosis , clinical characteristics and survival – findings from a UK population-based study. 2017;:1–5. doi:10.1111/bjh.14513

35 Redaniel MT, Martin RM, Ridd MJ, *et al.* Diagnostic intervals and its association with breast, prostate, lung and colorectal cancer survival in England: Historical cohort study using the Clinical Practice Research Datalink. *PLoS One* 2015;**10**:1–17. doi:10.1371/journal.pone.0126608

36 Obe EL. Myeloma Patient Experience Report 2016 Foreword. 2016.

37 Rubin GP, Saunders CL, Abel GA, *et al.* Impact of investigations in general practice on timeliness of referral for patients subsequently diagnosed with cancer : analysis of national primary care audit data. *Br J Cancer* 2015;**112**:676–87. doi:10.1038/bjc.2014.634

38 Barrett J, Hamilton W. Pathways to the diagnosis of lung cancer in the UK: a cohort study. *BMC Fam Pract* 2008;**9**:31. doi:10.1186/1471-2296-9-31

39 Jensen H, Tørring ML, Olesen F, *et al.* Cancer suspicion in general practice , urgent referral and time to diagnosis : a population-based GP survey and registry study. 2014.

40 Ades AE, Biswas M, Welton NJ, *et al.* Symptom lead time distribution in lung cancer: Natural history and prospects for early diagnosis. *Int J Epidemiol* 2014;**43**:1865–73. doi:10.1093/ije/dyu174

For peer review only - http://bmjopen.bmj.com/site/about/guidelines.xhtml

Table 1: Study characteristics

Study design	Study period	Population characteristics (age, gender)	Myeloma patients (Total)	Outcome measure (Interval)
**Friese et al 2009, (USA)** [8]				
Retrospective analysis	1992-2002	mean age 76.3<br>46% males	3831	Diagnostic
**Howell et al 2013, (UK)** [31]				
Survey	2004-2011	Median age 69.9<br>66.9% males	341	Patient<br>Diagnostic<br>Time to diagnosis
**Lyratzopoulos et al 2013, (UK)** [26]				
Audit data	2009-2010	Not reported	176	Primary Care
**Varga et al 2014, (Hungary)** [29]				
Retrospective analysis	Not reported	median age 60<br>50% males	193	Diagnostic
**Neal et al 2014, (UK)** [25]				
Retrospective analysis	2001-2002	mean age 72<br>53% males	221	Diagnostic
**Din et al 2015, (UK)** [24]				
Retrospective analysis	2007-2010	median age 72<br>56% males	500	Diagnostic
**Lyratzopoulos et al 2015, (UK)** [27]				
Audit data	2009-2010	Not reported	124	Patient
**Goldscbmidt et al 2016, (Israel)** [30]				
Retrospective analysis	2002-2011	median age 63<br>53% males	107	Diagnostic
**Swann et al 2017, (UK)** [28]				
Audit data	2014	Not reported	202	Primary<br>Diagnostic

For peer review only

For peer review only - http://bmjopen.bmj.com/site/about/guidelines.xhtml

Table 2: Symptoms and date definitions

Symptoms used	Onset of first symptom	Date of first Presentation in healthcare services	Date of first referral	Date of diagnosis
**Friese et al 2009, (USA)**				
Anaemia<br>Packed red blood cell transfusion<br>Back pain	N/A	1 year before diagnosis	N/A	SEER cancer diagnosis date
**Howell et al 2013, (UK)**				
Tiredness<br>Pain<br>Shortness of breath<br>Infections<br>Joint problems/Fractures<br>Stomach/bowel symptoms<br>Other	Patient reported	Patient reported	N/A	Date provided by the Haematological Malignancy Diagnostic service
**Lyratzopoulos et al 2013, (UK)**				
Not reported	Estimated based on patient's clinical records.	2 years before diagnosis	Date that the referral letter was sent	Clinical records and hospital correspondence
**Varga et al 2014, (Hungary)**				
Bone symptoms<br>Anaemia<br>Renal failure<br>General symptoms<br>Other<br>Tumour presence<br>Metastatic bone disease	N/A	3 years before diagnosis	N/A	Tertiary haematology centre
**Neal et al 2014, (UK)**				
Bleeding<br>Bone Pain<br>Bruising<br>Anaemia<br>Fatigue<br>Anorexia<br>Weight loss	N/A	1 year before diagnosis	N/A	First occurrence of a myeloma Read Code in the patient's record in CPRD database.
**Din et al 2015, (UK)**				
Bleeding<br>Bone Pain<br>Bruising<br>Anaemia<br>Fatigue<br>Anorexia<br>Weight loss	N/A	1 year before diagnosis	N/A	First occurrence of a myeloma Read Code in the patient's record in CPRD database.
**Lyratzopoulos et al 2015, (UK)**				
Not reported	N/A	2 years before	Date that the	Clinical records

For peer review only - http://bmjopen.bmj.com/site/about/guidelines.xhtml

		diagnosis	referral letter was sent	and hospital correspondence
**Goldscbmidt et al 2016, (Israel)***				
Pain (back, cervical spine, musculoskeletal, non-specific)<br>Infection<br>Weight loss<br>Fatigue<br>Peripheral edema<br>Constipation<br>Presyncope<br>Syncope<br>Dizziness	N/A	2 years before diagnosis	N/A	Israel National Cancer Registry
**Swann et al 2017, (UK)**				
Not reported	NA	2 years before diagnosis	Date that the referral letter was sent	Hospital Episode Statistics (HES)

For peer review only

Table 3: Length of intervals

Percentile	N	25th	50th	75th
**Patient Interval**				
Howell et al 2013	341	1	31	122
Lyratzopoulos et al 2015	124	0	13.5	31
Weighted estimate	465	0.7	26.3	97.7
**Primary care Interval**				
Lyratzopoulos et al 2013	176	5	21	55
Swann et al 2017	150	4.2	23.5	56.8
Weighted estimate	326	4.6	21.6	55.8
**Referral to Diagnosis interval**				
No papers reporting this interval				
**Diagnostic interval**				
Friese et al 2009	3831	27	99	252
Howell et al 2013	341	34	83	167
Varga et al, 2014	193	88	125	230
Neal et al, 2014	221	56	144	264
Din et al 2015	500	54	149	263
Goldscbmidt et al 2016	107	NR*	341	NR*
Swann et al 2017	202	24	53.5	107.5
Weighted estimate	5395	33.3	108.6	241.7
**Time to diagnosis**				
Howell et al, 2013	341	84	163	306

*Not reported

For peer review only - http://bmjopen.bmj.com/site/about/guidelines.xhtml

Titles and legends to figures

Figure 1

Title: Outcome definition

Figure 2

Title: Study selection flowchart

Figure 3

Title: Distribution of the intervals

Legend: Each circle corresponds to one study and the size is proportional to the total sample size. The blue diamond corresponds to the weighted estimate. For intervals with only one study (time to diagnosis) no weighted estimates were calculated. Y-axis corresponds to 1-Probability (interval > number of days) i.e. 0.25 corresponds to the 75th percentile and 0.75 to the 25th percentile.

For peer review only

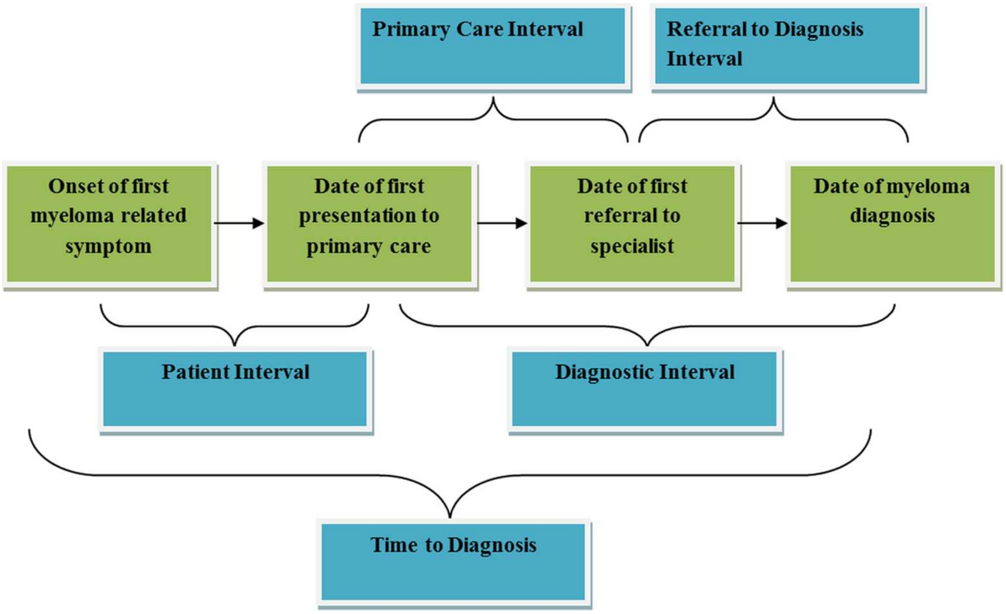

Figure 1: Outcome definition

108x65mm (300 x 300 DPI)

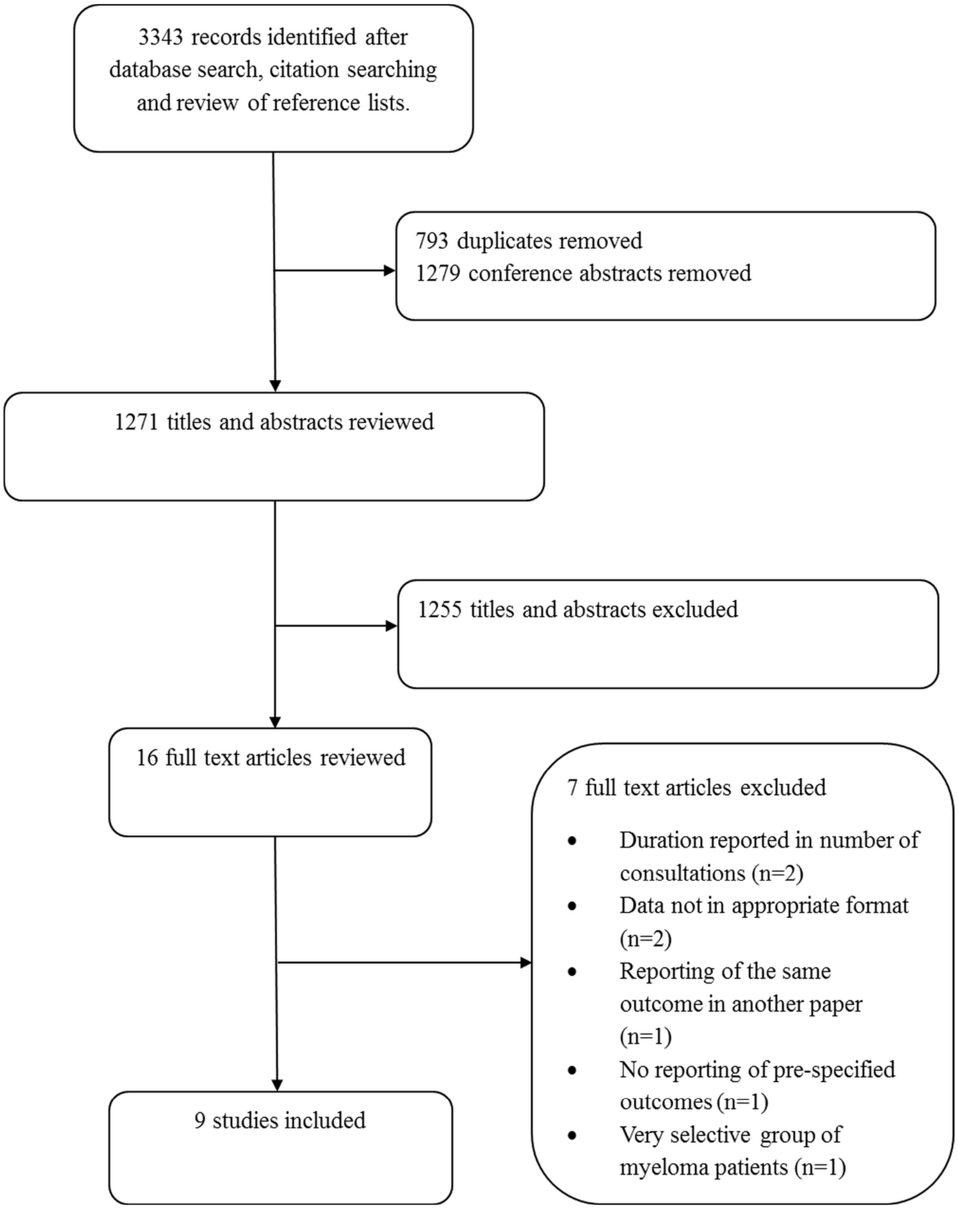

Figure 2: Study flowchart

214x270mm (300 x 300 DPI)

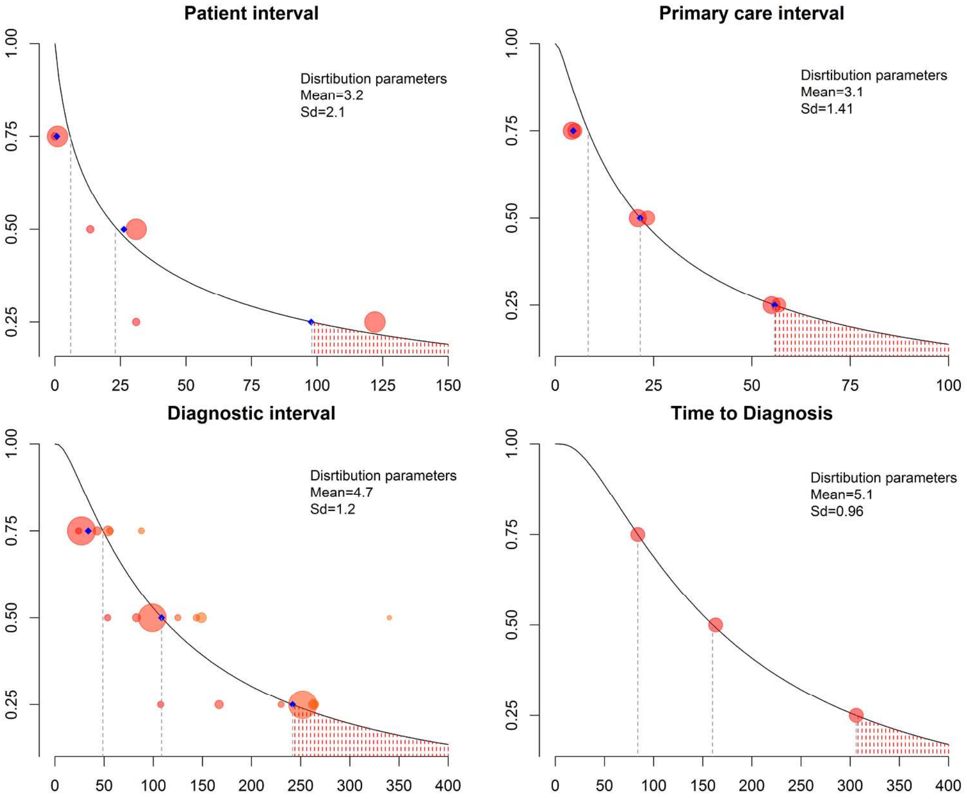

Figure 3: Distribution of the intervals. Each circle corresponds to one study and the size is proportional to the total sample size. The blue diamond corresponds to the weighted estimate. For intervals with only one study (time to diagnosis) no weighted estimates were calculated. Y-axis corresponds to 1-Probability (interval > number of days) i.e. 0.25 corresponds to the 75th percentile and 0.75 to the 25th percentile

237x189mm (300 x 300 DPI)

A1: Search strategy

1. Multiple Myeloma/
2. myeloma*.ti,ab.
3. 1 or 2
4. (time adj4 diagnos$).ti,ab.
5. (time adj4 consult$).ti,ab.
6. (time adj4 refer$).ti,ab.
7. (time adj4 present$).ti,ab.
8. 4 or 5 or 6 or 7
9. (delay$ adj4 diagnos$).ti,ab.
10. (delay$ adj4 consult$).ti,ab.
11. (delay$ adj4 refer$).ti,ab.
12. (delay$ adj4 present$).ti,ab.
13. (delay$ adj4 seek$).ti,ab.
14. (delay$ adj4 detect*).ti,ab.
15. 9 or 10 or 11 or 12 or 13 or 14
16. (interval adj4 consult$).ti,ab.
17. (interval adj4 consult$).ti,ab.
18. (interval adj4 refer$).ti,ab.
19. (interval adj4 present$).ti,ab.
20. 16 or 17 or 18 or 19
21. (late adj4 diagnosis).ti,ab.
22. (late adj4 detect*).ti,ab.
23. (late adj4 present$).ti,ab.
24. 21 or 22 or 23
25. diagnos$ delay$.ti,ab.
26. early diagnos$.ti,ab.
27. 8 or 15 or 20 or 24 or 25 or 26
28. 3 and 27

For peer review only

A2: Risk of bias summary

Legend: No / Yes / Not applicable

	1	2	3	4	5	6	7	8	9	10	11	12	13	14	15	16	17	18	19	20
Friese et al. 2009	Yes	Yes	Not applicable	Not applicable	No	Not applicable	No	Yes	Yes	No	Not applicable	Not applicable	Not applicable	Not applicable	Not applicable	Not applicable	Not applicable	Not applicable	Not applicable	Yes
Howell et al. 2013	Yes	Yes	No	Yes	Yes	Not applicable	No	Yes	Yes	Yes	No	Yes	No	No	Yes	Yes	No	Yes	Not applicable	Not applicable
Lyratzopoulos et al. 2013	Yes	Yes	Not applicable	Not applicable	Yes	Yes	No	Yes	Yes	Yes	Not applicable	Not applicable	Not applicable	Not applicable	Not applicable	Not applicable	Not applicable	Not applicable	Not applicable	Yes
Varga et al. 2014	Yes	No	Not applicable	Not applicable	No	Not applicable	No	Yes	Yes	No	Not applicable	Not applicable	Not applicable	Not applicable	Not applicable	Not applicable	Not applicable	Not applicable	No	Not applicable
Neal et al. 2014	Yes	Yes	Not applicable	Not applicable	Yes	Not applicable	Yes	Yes	Yes	Yes	Not applicable	Not applicable	Not applicable	Not applicable	Not applicable	Not applicable	Not applicable	Not applicable	Not applicable	Yes
Din et al. 2015	Yes	Yes	Not applicable	Not applicable	Yes	Not applicable	Yes	Yes	Yes	Yes	Not applicable	Not applicable	Not applicable	Not applicable	Not applicable	Not applicable	Not applicable	Not applicable	Not applicable	Yes
Lyratzopoulos et al. 2015	Yes	Yes	Yes	Yes	Yes	Not applicable	No	Yes	Yes	Yes	Not applicable	Not applicable	Not applicable	Not applicable	Not applicable	Not applicable	Not applicable	Not applicable	Not applicable	Yes
Goldscbmidt et al. 2016	Yes	Yes	Not applicable	Not applicable	Yes	Not applicable	No	Yes	Yes	No	Not applicable	Not applicable	Not applicable	Not applicable	Not applicable	Not applicable	Not applicable	Not applicable	Yes	Not applicable
Swann et al. 2017	Yes	Yes	Not applicable	Not applicable	Yes	Yes	No	Yes	Yes	Yes	Not applicable	Not applicable	Not applicable	Not applicable	Not applicable	Not applicable	Not applicable	Not applicable	Yes	Not applicable

A3: Risk of bias graph

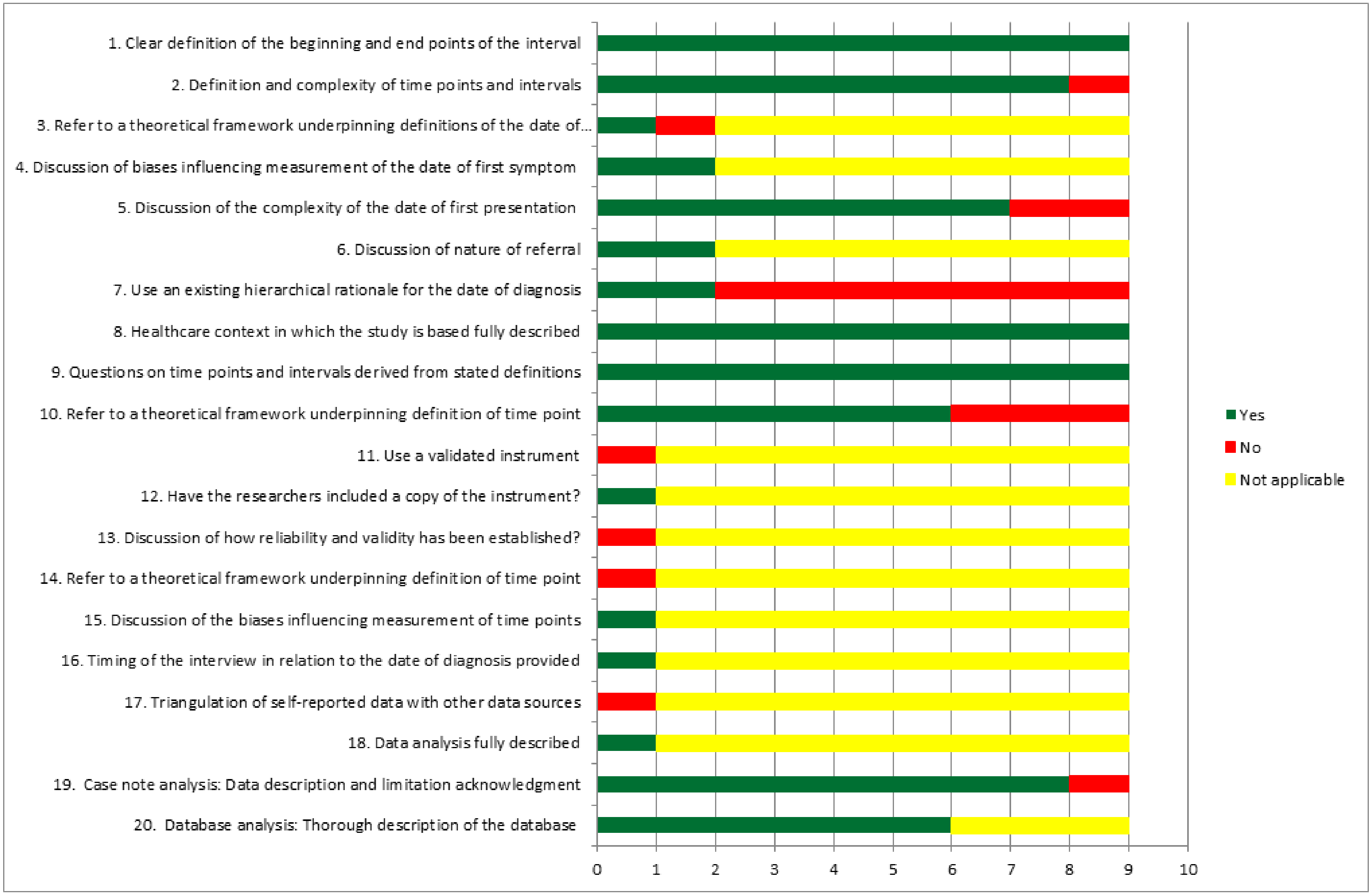

PRISMA 2009 Checklist

Section/topic	#	Checklist item	Reported on page #
**TITLE**			
Title	1	Identify the report as a systematic review, meta-analysis, or both.	1
**ABSTRACT**			
Structured summary	2	Provide a structured summary including, as applicable: background; objectives; data sources; study eligibility criteria, participants, and interventions; study appraisal and synthesis methods; results; limitations; conclusions and implications of key findings; systematic review registration number.	2
**INTRODUCTION**			
Rationale	3	Describe the rationale for the review in the context of what is already known.	4-5
Objectives	4	Provide an explicit statement of questions being addressed with reference to participants, interventions, comparisons, outcomes, and study design (PICOS).	5-6
**METHODS**			
Protocol and registration	5	Indicate if a review protocol exists, if and where it can be accessed (e.g., Web address), and, if available, provide registration information including registration number.	5-6
Eligibility criteria	6	Specify study characteristics (e.g., PICOS, length of follow-up) and report characteristics (e.g., years considered, language, publication status) used as criteria for eligibility, giving rationale.	5-6
Information sources	7	Describe all information sources (e.g., databases with dates of coverage, contact with study authors to identify additional studies) in the search and date last searched.	5-6
Search	8	Present full electronic search strategy for at least one database, including any limits used, such that it could be repeated.	p.5 & Appendix
Study selection	9	State the process for selecting studies (i.e., screening, eligibility, included in systematic review, and, if applicable, included in the meta-analysis).	6
Data collection process	10	Describe method of data extraction from reports (e.g., piloted forms, independently, in duplicate) and any processes for obtaining and confirming data from investigators.	6
Data items	11	List and define all variables for which data were sought (e.g., PICOS, funding sources) and any assumptions and simplifications made.	6
Risk of bias in individual studies	12	Describe methods used for assessing risk of bias of individual studies (including specification of whether this was done at the study or outcome level), and how this information is to be used in any data synthesis.	6
Summary measures	13	State the principal summary measures (e.g., risk ratio, difference in means).	6
Synthesis of results	14	Describe the methods of handling data and combining results of studies, if done, including measures of consistency (e.g., I^2) for each meta-analysis.	6

For peer review only - http://bmjopen.bmj.com/site/about/guidelines.xhtml

PRISMA 2009 Checklist

Section/topic	#	Checklist item	Reported on page #
Risk of bias across studies	15	Specify any assessment of risk of bias that may affect the cumulative evidence (e.g., publication bias, selective reporting within studies).	6-7
Additional analyses	16	Describe methods of additional analyses (e.g., sensitivity or subgroup analyses, meta-regression), if done, indicating which were pre-specified.	6-7
**RESULTS**			
Study selection	17	Give numbers of studies screened, assessed for eligibility, and included in the review, with reasons for exclusions at each stage, ideally with a flow diagram.	7 (figure 1)
Study characteristics	18	For each study, present characteristics for which data were extracted (e.g., study size, PICOS, follow-up period) and provide the citations.	7 (table 1)
Risk of bias within studies	19	Present data on risk of bias of each study and, if available, any outcome level assessment (see item 12).	Appendix
Results of individual studies	20	For all outcomes considered (benefits or harms), present, for each study: (a) simple summary data for each intervention group (b) effect estimates and confidence intervals, ideally with a forest plot.	p. 7-9<br>table 2, figure 3
Synthesis of results	21	Present results of each meta-analysis done, including confidence intervals and measures of consistency.	p. 7-9<br>table 2, figure 3
Risk of bias across studies	22	Present results of any assessment of risk of bias across studies (see Item 15).	8-9 appendix
Additional analysis	23	Give results of additional analyses, if done (e.g., sensitivity or subgroup analyses, meta-regression [see Item 16]).	9
**DISCUSSION**			
Summary of evidence	24	Summarize the main findings including the strength of evidence for each main outcome; consider their relevance to key groups (e.g., healthcare providers, users, and policy makers).	9-10
Limitations	25	Discuss limitations at study and outcome level (e.g., risk of bias), and at review-level (e.g., incomplete retrieval of identified research, reporting bias).	10-12
Conclusions	26	Provide a general interpretation of the results in the context of other evidence, and implications for future research.	10-13
**FUNDING**			

For peer review only

PRISMA 2009 Checklist

Funding	27	Describe sources of funding for the systematic review and other support (e.g., supply of data); role of funders for the systematic review.	14

From: Moher D, Liberati A, Tetzlaff J, Altman DG, The PRISMA Group (2009). Preferred Reporting Items for Systematic Reviews and Meta-Analyses: The PRISMA Statement. PLoS Med 6(7): e1000097. doi:10.1371/journal.pmed1000097

For more information, visit: **www.prisma-statement.org**.

For peer review only

For peer review only - http://bmjopen.bmj.com/site/about/guidelines.xhtml

MOOSE Checklist for Meta-analyses of Observational Studies

Item No	Recommendation	Reported on Page No
Reporting of background should include		
1	Problem definition	P4
2	Hypothesis statement	P5
3	Description of study outcome(s)	P5
4	Type of exposure or intervention used	NA
5	Type of study designs used	P5
6	Study population	P5
Reporting of search strategy should include		
7	Qualifications of searchers (eg, librarians and investigators)	P5
8	Search strategy, including time period included in the synthesis and key words	P5 & appendix
9	Effort to include all available studies, including contact with authors	P6
10	Databases and registries searched	P5
11	Search software used, name and version, including special features used (eg, explosion)	P5 & appendix
12	Use of hand searching (eg, reference lists of obtained articles)	NA
13	List of citations located and those excluded, including justification	P7/F2
14	Method of addressing articles published in languages other than English	P5
15	Method of handling abstracts and unpublished studies	P5
16	Description of any contact with authors	P6
Reporting of methods should include		
17	Description of relevance or appropriateness of studies assembled for assessing the hypothesis to be tested	P5
18	Rationale for the selection and coding of data (eg, sound clinical principles or convenience)	P5-6
19	Documentation of how data were classified and coded (eg, multiple raters, blinding and interrater reliability)	P5-6
20	Assessment of confounding (eg, comparability of cases and controls in studies where appropriate)	NA
21	Assessment of study quality, including blinding of quality assessors, stratification or regression on possible predictors of study results	P 6-7
22	Assessment of heterogeneity	P 7-8, 10
23	Description of statistical methods (eg, complete description of fixed or random effects models, justification of whether the chosen models account for predictors of study results, dose-response models, or cumulative meta-analysis) in sufficient detail to be replicated	P 7-8
24	Provision of appropriate tables and graphics	T1,T2,T3, F1, F2, F3
Reporting of results should include		
25	Graphic summarizing individual study estimates and overall estimate	T2, F3
26	Table giving descriptive information for each study included	T1
27	Results of sensitivity testing (eg, subgroup analysis)	P9, 11
28	Indication of statistical uncertainty of findings	P10

For peer review only

Item No	Recommendation	Reported on Page No
Reporting of discussion should include		
29	Quantitative assessment of bias (eg, publication bias)	P8
30	Justification for exclusion (eg, exclusion of non-English language citations)	NA
31	Assessment of quality of included studies	P8, appendix
Reporting of conclusions should include		
32	Consideration of alternative explanations for observed results	P10-11
33	Generalization of the conclusions (ie, appropriate for the data presented and within the domain of the literature review)	P11-12
34	Guidelines for future research	P12
35	Disclosure of funding source	P14

From: Stroup DF, Berlin JA, Morton SC, et al, for the Meta-analysis Of Observational Studies in Epidemiology (MOOSE) Group. Meta-analysis of Observational Studies in Epidemiology. A Proposal for Reporting. *JAMA*. 2000;283(15):2008-2012. doi: 10.1001/jama.283.15.2008.

Transcribed from the original paper within the NEUROSURGERY® Editorial Office, Atlanta, GA, United Sates. August 2012.

For peer review only

BMJ Open

Quantifying intervals to diagnosis in myeloma: a systematic review and meta-analysis

Journal:	*BMJ Open*
Manuscript ID	bmjopen-2017-019758.R2
Article Type:	Research
Date Submitted by the Author:	12-Apr-2018
Complete List of Authors:	Koshiaris, Constantinos; University of Oxford Department of Primary Care Health Sciences<br>Oke, Jason; University of Oxford, Nuffiled Department of Primary Care Health Sciences<br>Abel, Lucy; University of Oxford, Nuffield Department of Primary Care Health Sciences<br>Nicholson, Brian; University of Oxford, Nuffield Dept Primary Care Health Sciences<br>Ramasamy, Karthik ; Churchill Hospital, Department of Haematology, Oxford University Hospitals NHS Trust<br>van den Bruel, Ann; University of Oxford, Dept of Primary Health Care
**Primary Subject Heading**:	Haematology (incl blood transfusion)
Secondary Subject Heading:	Health services research
Keywords:	Myeloma < HAEMATOLOGY, time to diagnosis, early diagnosis, PRIMARY CARE, systematic review, delays

SCHOLARONE™
Manuscripts

Quantifying intervals to diagnosis in myeloma: a systematic review and meta-analysis

Constantinos Koshiaris[1] MSc, Jason Oke[1] PhD, Lucy Abel[1] MSc, Brian D Nicholson[1] MRCGP, Karthik Ramasamy[2 3] PHD, Ann Van den Bruel PHD[1]

[1]Nuffield Department of Primary Care Health Sciences, University of Oxford, Radcliffe Primary Care Building, Radcliffe Observatory Quarter, Woodstock Road, Oxford OX2 6GG

[2] Department of Haematology, Oxford University Hospitals NHS Trust, Old Road, Headington, Oxford OX3 7LE

[3] NIHR BRC Blood Theme, Oxford

Corresponding author:

Mr. Constantinos Koshiaris (constantinos.koshiaris@phc.ox.ac.uk)

Telephone number: 01865 289317

Nuffield Department of Primary Care Health Sciences, University of Oxford, Radcliffe Primary Care Building, Radcliffe Observatory Quarter, Woodstock Road, Oxford OX2 6GG

Word count: 3810

Key words: multiple myeloma, time to diagnosis, early diagnosis, primary care, systematic review, delays

For peer review only

For peer review only - http://bmjopen.bmj.com/site/about/guidelines.xhtml

ABSTRACT

Objectives

To quantify the duration of each step of the diagnostic pathway for multiple myeloma patients from symptom onset to confirmation of diagnosis

Design

Systematic review and meta-analysis

Data sources and selection criteria

The MEDLINE and EMBASE databases were searched up until January 2018 to identify articles which reported time intervals from onset of symptoms to diagnosis. Articles focusing on children or adolescents and on the asymptomatic form of the disease (monoclonal gammopathies and smouldering myeloma) were excluded.

Data collection and data analysis

Data were extracted independently by two reviewers. Weighted estimates of the median and interquartile range were calculated. Risk of bias was assessed using the Aarhus checklist.

Main results

Nine studies were included. The patient interval (first symptom – first presentation) has a median of 26.3 days (IQR: 1 to 98, n=465, 2 studies). Subsequently, the primary care (first presentation – first referral) interval is 21.6 days (IQR: 4.6 to 55.8, n=326, 2 studies), the diagnostic interval (first presentation – diagnosis) 108.6 days (IQR: 33.3 to 241.7, n=5395, 7 studies) and the time to diagnosis (first symptom – diagnosis) interval 163 days (IQR: 84 to 306, n=341, 1 study). No studies reported data for the referral to diagnosis interval.

Conclusion

The review demonstrates that there is scope for significant reductions in the time to myeloma diagnosis. At present, many patients experience a diagnostic interval longer than 3 months until diagnosis is confirmed.

For peer review only - http://bmjopen.bmj.com/site/about/guidelines.xhtml

Review registration

Not available. Protocol available from authors

Strengths and limitations of this study

Strengths

- First systematic review that quantified the whole diagnostic pathway for multiple myeloma patients including the different intervals in each step of the pathway
- Use of all available information including the interquartile range rather than just focusing on measures of central tendency like the mean and the median

Limitations

- No universally accepted methods for formal meta-analysis of median and interquartile range
- Limited number of studies reporting the patient and primary care intervals and no studies were reporting the referral to diagnosis interval so any inferences regarding the referral to diagnosis interval should be interpreted with caution.

For peer review only - http://bmjopen.bmj.com/site/about/guidelines.xhtml

INTRODUCTION

Myeloma is a haematological malignancy characterised by uncontrolled plasma cell production in the bone marrow. It was the 17th most common cancer in the UK in 2013 accounting for 2% of all new cancer cases. Currently there are more than 17500 myeloma patients in the UK with approximately 5500 cases being diagnosed every year[1,2]. It is a cancer that mainly affects the elderly population with 59% of the patients being diagnosed over the age of 70[2] and with a 5 year survival of 47%[3].

It is considered one of the hardest cancers to suspect in primary care. Symptoms of myeloma are very common in other conditions as well, such as back pain, bone pain, fatigue and repeated infections[4]. This in combination with the fact that myeloma is a very rare condition in primary care results in very low predictive values for individual symptoms. For example, primary care patients with back pain, which is one of the most common myeloma symptoms, only have a 0.1% risk of myeloma[5]. By comparison, patients with rectal bleeding have a 2.4% risk of colorectal cancer[6].

As a result, half of symptomatic myeloma patients have three or more consultations in primary care before they are referred to specialist care which is more than in any other cancer[7]. Attributing symptoms to comorbidities further prolongs the diagnostic process, which is particularly relevant in this older age group [8,9].

Delays in diagnosing myeloma allow complications to develop (end organ damage), such as pathological fractures, irreversible renal failure and in some cases spinal cord compression[10–12]. These are considered medical emergencies in their own right and limit the opportunity for applying effective treatment[13]. A delayed diagnosis is also linked with higher cancer stage[14,15] which is in turn associated with poorer survival[16]. Patients with

For peer review only - http://bmjopen.bmj.com/site/about/guidelines.xhtml

longer diagnostic intervals also experience shorter disease free survival and more complications from treatment[14].

Quantifying the time-intervals leading up to diagnosis is important as it will inform future interventions that aim to shorten the diagnostic process. The aim of this systematic review was to quantify each step of the diagnostic pathway to myeloma diagnosis and identify where to focus efforts to reduce diagnostic delay.

METHODS

A protocol is available in the appendix (A1). A copy of the search strategy can be seen in the appendix (A2). We searched EMBASE and MEDLINE until January 2018 for studies that quantified any or all of the following five intervals[17]: the patient interval (from symptom onset to first consultation); the primary care interval (from first consultation for that symptom to referral to secondary care); the diagnostic interval (from first consultation with a myeloma related symptom to diagnosis) and the time to diagnosis (from symptom onset to diagnosis). In addition we looked for studies that were estimating the Referral to diagnosis interval (Figure 1). Citation searching of key references like the Aarhus statement was conducted and we also searched the reference list of systematic reviews with similar research questions [18,19]. We included any study designs that quantified at least one of the intervals mentioned above in days or months. Studies reporting the length of an interval only in number of consultations or referrals were excluded as were studies focusing on children or adolescents (< 18 years) and on the asymptomatic forms of the disease (monoclonal gammopathies and smouldering myeloma). We included only papers with an abstract in English, but did not exclude full text articles based on language (as long as there was an English abstract). Conference abstracts were excluded. Two reviewers (CK/LA) selected papers for inclusion using the criteria listed above, on title and abstracts first and on full text second. Disagreements were resolved through discussion with a third reviewer (JO/AVB).

For peer review only - http://bmjopen.bmj.com/site/about/guidelines.xhtml

For peer review only

Data extraction

Two reviewers (CK/LA) independently extracted data from the included studies into a pre-defined spreadsheet. Study characteristics including author, year of publication, country of data collection, type of study, myeloma related symptoms and sample size were extracted, as well as descriptive statistics including median, interquartile range, range, mean and the standard deviation (SD) for each interval. Authors were contacted if data were not available or not in the appropriate format for extraction (i.e. categorical rather than continuous).

Risk of bias assessment

The risk of bias was assessed by two independent researchers (CK/LA) using the Aarhus checklist[18]. The Aarhus checklist is a 20 item tool designed to help researchers design and evaluate studies on early diagnosis of cancer. It examines studies in terms of acknowledgment of the different biases influencing time point measurement and interval definition.

Analysis

In the context of illness duration, intervals are usually not normally distributed, therefore we used the median and interquartile-range (IQR) to summarise the data. We present the 25th, 50th and 75th percentiles for all intervals. For intervals reported by more than one study, the pooled estimate was calculated by taking a weighted mean for each percentile and the weight was obtained by dividing the sample size in each study with the total numbers of patients. We also fitted a distribution through the 3 weighted percentile estimates where appropriate in order to try and generate the shape of the distribution of the interval under investigation. We chose the lognormal distribution as time intervals are usually skewed to the right[4]. A pre-specified sensitivity analysis was conducted by excluding the study with the higher risk of bias. The sensitivity analysis was conducted only for the diagnostic interval as the rest of the outcomes were reported by only 1 or 2 papers which can be seen in table 1.

For peer review only - http://bmjopen.bmj.com/site/about/guidelines.xhtml

Patient and public involvement

No patients or public were involved in this study

RESULTS

We identified 3343 citations from the EMBASE and MEDLINE searches. After removal of conference abstracts and duplicates, we screened 1271 titles and abstracts and 16 studies were candidate for inclusion. Nine studies were included in the final analysis (Figure 2). Seven papers in total were excluded for the following reasons: two because they were reporting the duration in numbers of consultations [5,7]; one because none of the pre-specified outcomes were reported[20]; one reported the same outcome based on the same database as one of the other included papers so inclusion of this paper in the data synthesis would result in double counting [21]; two papers because data were not in an appropriate format [14,22]; one because the interval under investigation was reported only for patients that were referred to very specific specialisations making it a very selective population compared to the other studies [23]

Study Characteristics

A summary of all the included papers is provided in table 1. Studies were published between 2009 and 2018 and the sample size ranged from 107 to 3831 patients. Five studies reported intervals in various cancers[24–28] ; three reported only myeloma[8,29,30]; and one for haematological malignancies[31].

Six studies were conducted in the UK[24–28,31], one in the USA[8], one in Hungary[29] and one in Israel[30]. Two UK studies used data from two separate Clinical Practice Research Datalink (CPRD) cohorts[24,25], a database of routinely collected electronic primary care records. Two other UK studies used data from the English National Audit of Cancer Diagnosis in Primary Care 2009-2010[32]: we extracted primary care interval data from the

For peer review only - http://bmjopen.bmj.com/site/about/guidelines.xhtml

larger study and the patient interval from the smaller study[26,27]. Another UK study used data from the English National Audit of Cancer Diagnosis in Primary care 2014 and the last UK study was a patient survey on patients diagnosed with haematological malignancies[28,31]. The study conducted in Hungary analysed data collected from patients treated in a haematology centre and the study conducted in the USA analysed a retrospective database collected from the Surveillance, Epidemiology, and End Results Program (SEER) program[8,29]. The study conducted in Israel used data from the Israeli health maintenance (HMO) organisation linked with the Israel National Cancer Registry (NCR) [30].

Definition of intervals to diagnosis

There was substantial heterogeneity in the symptoms and time points used to define each interval (table 2). In total, 19 different symptoms were used to define the start of myeloma but studies varied greatly regarding which symptom (or symptoms) was used, ranging from 3 to a maximum of 12 symptoms. Three studies did not report the starting symptoms [26–28]. Also some studies included multiple symptoms in more general categories[24,25,31]. For example, Howell et al used a general pain category which included musculoskeletal, abdominal, chest and other type of pains while the two CPRD studies included multiple musculoskeletal symptoms under a general bone pain category[24,25]. In addition studies using CPRD or SEER data were using predefined symptoms to identify the onset of disease while other studies like Howell et al. documented the full range of symptoms reported by the patients during this time.

The start of the measuring period was defined as the date of onset of the first symptom or the date of first presentation for a myeloma related symptom depending on whether the studies were investigating the patient interval, the diagnostic or both. The authors of the studies used various pre diagnostic time intervals to identify the first symptom (at one, two or three years

For peer review only - http://bmjopen.bmj.com/site/about/guidelines.xhtml

before diagnosis). Three identified the first symptom at one year before diagnosis, three at two years and one at three years. One study used patient reported dates. Goldschmidt N et al [30] did not use the first symptom as the start of the measurement period but they used the first combination of symptom and laboratory result (i.e. the earliest of blood test + pain complaint or two blood tests within a month or two pain complaints within 1-3 months).

Risk of Bias

Most of the studies included in the analysis had a low risk of bias (Appendix A3, A4). All studies clearly defined the start and end point of the intervals and in most cases there was an adequate description of the databases along with the strengths, limitations and biases arising from the definitions of the different intervals and time point. Only one study did not mention the different limitations and biases arising from the study design and the choice of definitions for time points and intervals[29]. Most common sources of bias that were described included recall bias for studies that were using patient reported data and misclassification bias for studies that were using databases like CPRD. Most studies used a theoretical framework to define each interval usually the one reported by Olesen et al[17] or the Aarhus statement[18]. The category with the higher risk of bias was the use of a hierarchical rationale to determine the date of diagnosis i.e. date of first histological confirmation of the malignancy or date of admission to the hospital for example. Most studies mentioned how the date of diagnosis was obtained but there was no adequate description on how the choice of a particular definition can affect the diagnostic pathway.

Quantifying intervals

Seven papers reported the diagnostic interval [8,24,25,28–31], two the patient interval[27,31], two the primary care interval[26,28], and one the time to diagnosis interval[31]. No studies reported the referral to diagnosis interval. The length of the different intervals can be seen in

For peer review only - http://bmjopen.bmj.com/site/about/guidelines.xhtml

table 3 and the fitted log normal distributions in figure 3 along with the parameters used to fit them.

For the diagnostic interval, the pooled weighted mean of the 50th percentile is 108.6 days (n=5398) and the IQR is from 33.3 to 241.7 (n=5288). Removing the study with the largest risk of bias[29] based on the Aarhus statement checklist did not alter the results (107.9 days, IQR: 31.3-242.2). An additional sensitivity analysis was conducted by excluding the Goldschmidt N et al [30] study as it was an outlier but the results were not affected (pooled median of 103.8). While all the studies reported a median diagnostic interval less than 5 months this study had an interval of 11.2 months. The IQR was not estimated for this sensitivity analysis as it was not reported by the authors.

The pooled estimate of the 50th percentile of the patient interval is 26.3 days (IQR: 0.7-97.7, n=465). The primary care interval was reported by two studies [26,28] with a median of 21.6 days (IQR: 4.6-55.8, n=326) and the time to diagnosis interval was also reported by one study[31] with a median of 163 days (IQR: 84-306, n=341).

No study reported the referral to diagnosis interval but it can be inferred by subtracting the median length of the primary care and patient intervals from the diagnostic interval or the time to diagnosis interval which suggests that the median length of the referral to diagnosis interval can range from 60.7 to 115.1 days depending on whether we use the diagnostic or the time to diagnosis interval for the inference.

DISCUSSION

Our results show that myeloma patients experience symptoms for a median of approximately one month before seeking help and 25% of patients wait for more than three months (98 days). After attending primary care with symptoms, the median time to diagnosis is 108.6

For peer review only - http://bmjopen.bmj.com/site/about/guidelines.xhtml

days (IQR: 33.3-241.7) with 25% of patients waiting longer than eight months. No studies report the referral to diagnosis interval

Strengths and weaknesses

This is the first systematic review that quantified the patient pathway of myeloma from onset of first symptom to diagnosis. There were no restrictions in the search strategy in terms of study design or health care systems. We focused our search on two medical databases that are more likely to contain papers on diagnostic pathways but we acknowledge that this might have affected the identification of all literature. To counter this, we included additional strategies like citation searching of some key references and searching the reference lists of similar reviews.

We excluded conference abstracts, although there were several that addressed the review question. The reason for this is that the length of the different intervals reported is affected by design decisions like the choice of when to start the measurement period, initial symptoms, data collection methods etc. Conference abstracts do not report this level of detail in their methods, and therefore could not be included.

In addition to measures of central tendency like the median we included the 25th and 75th percentiles, which are particularly important since time interval data are skewed to the right. Examining all three can provide a more complete idea of the delays that the patients experience especially at the tails of the distribution. Measures of central tendency like the mean might not be the most appropriate to describe the distribution as they tend to be overestimated when used on positively skewed distributions which would also make comparison with other cancer intervals more difficult as they are usually quantified using the median and IQR.

For peer review only - http://bmjopen.bmj.com/site/about/guidelines.xhtml

For peer review only

The main limitation of the analysis is that currently there are no universally accepted formal methods to perform meta-analysis of medians and interquartile ranges. To overcome this, we combined estimates of the percentiles after weighting them based on the sample size: a method equivalent to a fixed effects meta-analysis. Our estimates of the diagnostic interval might therefore be an underestimation, due to the fact that the biggest study reported one the lowest diagnostic intervals [8]. This however does not change the interpretation of the results as these estimates still suggest very long diagnostic intervals for myeloma patients. We were not able to produce a confidence interval around the median and interquartile range as these are not usually measures that are reported by the included studies, thus we present only the point estimate of each percentile. In addition there are no formal ways of estimating statistical heterogeneity when meta-analysing median and IQR.

No studies reported the referral to diagnosis interval and it was inferred based on the results of the other interval so our results regarding this interval should be interpreted with a lot of caution.

Sources of heterogeneity

As mentioned in the strengths and limitations no formal ways of estimating heterogeneity currently exist when performing meta-analysis of medians and IQR. In order to get an approximate measure of heterogeneity we also performed a meta-analysis of the means for which we had confidence intervals or we could approximate (appendix A5) which resulted in an I-squared statistics of 98.6% (diagnostic interval). Although we expect high heterogeneity due to various design decisions which are described below, this statistic should be interpreted with a lot of caution as it might be an overestimation. We believe that to be the case because of the very small uncertainty for each within-study estimate. This results in very narrow confidence intervals around each study which do not overlap and thus artificially inflate the I-

For peer review only - http://bmjopen.bmj.com/site/about/guidelines.xhtml

For peer review only

squared statistic. In addition for three out of seven studies either the means or the confidence intervals had to be approximated which could potentially be introducing more bias on the effect and heterogeneity estimates. Heterogeneity estimates might have been different if we were able to obtain confidence intervals around median and IQR. We believe that clinical heterogeneity is more important in this case.

In order to compute intervals, the definition of the beginning and the end of the interval is crucial. There was variability in how studies defined starting points, especially for the first symptom and the first presentation to healthcare, using medical records or patient recall. Studies that use patient reported outcomes tend to suffer from recall bias which might lead to overestimation or underestimation of the different intervals while studies using medical records tend to suffer from loss to follow up and misclassification.

For studies that were using electronic health records there was no agreement on when exactly myeloma starts to manifest. The time used to detect related symptoms prior to diagnosis spanned from one to three years. Although most studies used one year before diagnosis, there is some evidence to suggest that symptoms might be present for more than one year[33] which may have led to an underestimation of intervals in these studies. On the other hand, the more you extend the symptom period the more likely you are to detect symptoms that are unrelated to myeloma which leads to the overestimation of the length of the intervals, especially with symptoms that are so aspecific such as back pain. In order to explore this we conducted a sensitivity analysis where we estimated the length of the diagnostic interval by stratifying according to the time used to define the presenting symptoms with studies going one year back having a median of 105 days vs. more than one year back having a median of 142.3 day which might be explaining some of the observed variability.

For peer review only - http://bmjopen.bmj.com/site/about/guidelines.xhtml

For peer review only

Even though there are various sources of heterogeneity all the sensitivity analyses that were conducted were not changing the result trend and their interpretation, as almost all studies were reporting diagnostic intervals longer than three months irrespectively of the way the study was conducted.

Findings compared to existing research

Our estimate for the patient interval is in-line with the findings of another study[22] which reported that 15% of myeloma patients wait more than three months before they go to the doctor. This study explained this delay in terms of patients' lack of understanding of the seriousness of their symptoms because of their non-specific nature.

Myeloma patients experience the longest primary care interval out of all cancers with a median of 21.6 days. Other cancers that have been shown to have long primary care intervals include renal and lung with a median of 14 days [28]. The long primary care interval for myeloma patients could be explained by the fact that symptoms on their own are not enough for referral and multiple blood tests need to be conducted like the full blood count, calcium, creatinine and inflammatory markers. Conducting multiple tests has been shown to extent the primary care interval [34].

The diagnostic interval, which takes place in health care and could potentially be amenable to improvement, is longer for myeloma than for many other cancers. It has been shown that only 17.2% of myeloma patients are referred through the suspected cancer referral pathway ("two-week" wait) which is lower than other cancers like breast for example which is approximately 43% [20,35]. This could be due to the non-specific nature of the symptoms which make it hard for both the GP and the patient to suspect the presence of myeloma. This might also explain the difference in the length of the diagnostic intervals between these two cancers as the median diagnostic interval for breast cancer is approximately 14 days [36].

For peer review only - http://bmjopen.bmj.com/site/about/guidelines.xhtml

Other cancers with a similarly long median diagnostic interval also have non-specific clinical presentations, such as lung cancer which has a diagnostic interval of 88 days[36].

Implications for future research

We were not able to estimate the referral to diagnosis care interval directly although it is reasonable to believe that it might be longer than the primary care. As in other cancers referrals to different specialties or with an insufficient level of urgency, or multiple referrals can prolong the referral to diagnosis interval. [37]. The choice of referral route has also been shown to be a strong predictor of the length of the diagnostic interval i.e. patients that are diagnosed thought a referral pathway for cancer tend to have shorter intervals [38]. Future studies should not only estimate the duration of primary and referral to diagnosis intervals, but also investigate the impact of one setting on the other as in most cases the type and severity of symptoms will determine the speciality and urgency of referral.

Also it is still not clear how long before diagnosis myeloma symptoms start to occur. In lung cancer, studies on symptom lead time (the time between symptoms attributable to cancer and diagnosis) show that symptom incidence increases considerably 6 months before diagnosis but no such study has been conducted for myeloma[39].

CONCLUSION

Myeloma is a complex disease to diagnose due to a combination of different factors. Firstly, myeloma symptoms (like back pain and fatigue) are common and mostly caused by benign conditions, resulting in patients not visiting their doctor and in combination with the rarity of the disease, making it hard for GPs to suspect this cancer. In addition there is no effective screening as this might result in people having a lot of unnecessary tests and potentially over diagnosing MGUS thus any benefits from the screening programme cannot outweigh the

For peer review only - http://bmjopen.bmj.com/site/about/guidelines.xhtml

cost. Due to the above myeloma patients tend to experience long diagnostic intervals and our results indicate that in some cases it can be over eight months. There is potential for meaningful reductions in the time to diagnosis especially for the diagnostic interval which could improve patient outcomes but more research is required in order to do that. Further and more in-depth exploration of the diagnostic pathway is required especially for the intervals we were not able to explore in this study like the referral to diagnosis interval and its link with the primary care interval and development of interventions that aim to reduce the length of the diagnostic interval.

FUNDING

This manuscript presents work carried out as part of a DPhil scholarship awarded to Mr Constantinos Koshiaris funded by the Primary Care Research Trust, the university of Oxford and NIHR Oxford CLAHRC. AVDB is supported through the National Institute for Health Research (NIHR) Diagnostic Evidence Co-operative Oxford at Oxford Health Foundation Trust (IS_DEC_0812_100).

The research was supported by the National Institute for Health Research (NIHR) Collaboration for Leadership in Applied Health Research and Care Oxford at Oxford Health NHS Foundation Trust. The views expressed are those of the authors and not necessarily those of the NHS, the NIHR or the Department of Health.

For peer review only - http://bmjopen.bmj.com/site/about/guidelines.xhtml

ACKNOWLEDGMENTS

This study has been presented as an oral presentation at the SAPC conference, Dublin July 2016

Six authors contributed to this study: Constantinos Koshiaris MSc, Jason Oke PhD, Lucy Abel MSc, Brian D Nicholson MRCGP, Karthik Ramasamy PhD, and Ann Van den Bruel PhD. CK designed the study with input from AVDB and JO. All authors were involved in the conduct of the study, interpreting the results, and in revising and correcting the manuscript. CK and LA reviewed and extracted the data from articles. CK and JO planned and conducted the analysis. The manuscript drafting was led by CK with the contribution of all authors. All authors read and approved the final version of the manuscript.

CONFLICT OF INTEREST

No authors report a conflict of interest

DATA SHARING STATEMENT

The dataset is available on request from the corresponding author.

For peer review only

REFERENCES

1 England PH. National Cancer Intelligence Network Cancer statistics : availability and location About Public Health England. 2015.

2 Myeloma statistics | Cancer Research UK. http://www.cancerresearchuk.org/health-professional/cancer-statistics/statistics-by-cancer-type/myeloma#heading-Six (accessed 26 Aug 2016).

3 Quaresma M, Coleman MP, Rachet B. 40-year trends in an index of survival for all cancers combined and survival adjusted for age and sex for each cancer in England and Wales , 1971 – 2011 : a population-based study. *Lancet* 2015;**385**:1206–18. doi:10.1016/S0140-6736(14)61396-9

4 Lyratzopoulos G, Wardle J, Rubin G. Rethinking diagnostic delay in cancer: how difficult is the diagnosis? *BMJ* 2014;**349**:g7400–g7400. doi:10.1136/bmj.g7400

5 Shephard EA, Neal RD, Rose P, *et al.* Quantifying the risk of multiple myeloma from symptoms reported in primary care patients: a large case-control study using electronic records. *Br J Gen Pr* 2015;**65**:e106-13. doi:10.3399/bjgp15X683545

6 Hamilton W, Round A, Sharp D, *et al.* Clinical features of colorectal cancer before diagnosis : a population-based case – control study. 2005;:399–405. doi:10.1038/sj.bjc.6602714

7 Lyratzopoulos G, Neal RD, Barbiere JM, *et al.* Variation in number of general practitioner consultations before hospital referral for cancer: Findings from the 2010 National Cancer Patient Experience Survey in England. *Lancet Oncol* 2012;**13**:353–65. doi:10.1016/S1470-2045(12)70041-4

8 Friese CR, Abel G a., Magazu LS, *et al.* Diagnostic delay and complications for older adults with multiple myeloma. *Leuk Lymphoma* 2009;**50**:392–400. doi:10.1080/10428190902741471

9 Macdonald S, Macleod U, Campbell NC, *et al.* Systematic review of factors influencing patient and practitioner delay in diagnosis of upper gastrointestinal cancer. *Br J Cancer* 2006;**94**:1272–80. doi:10.1038/sj.bjc.6603089

10 Tosi P. Diagnosis and treatment of bone disease in multiple myeloma: spotlight on spinal involvement. *Scientifica (Cairo)* 2013;**2013**:104546. doi:10.1155/2013/104546

11 Sonmez M, Akagun T, Topbas M, *et al.* Effect of pathologic fractures on survival in multiple myeloma patients : a case control study. 2008;**4**:1–4. doi:10.1186/1756-9966-27-Received

12 Wirk B. Renal failure in multiple myeloma: a medical emergency. *Bone Marrow Transplant* 2011;**46**:771–83. doi:10.1038/bmt.2011.8

13 Smith D, Yong K. Multiple myeloma. *BMJ* 2013;**346**:f3863–f3863. doi:10.1136/bmj.f3863

14 Kariyawasan CC, Hughes D a., Jayatillake MM, *et al.* Multiple myeloma: causes and consequences of delay in diagnosis. *Qjm* 2007;**100**:635–40.

For peer review only - http://bmjopen.bmj.com/site/about/guidelines.xhtml

doi:10.1093/qjmed/hcm077

15 Ong F, Hermans J, Noordijk EM, *et al.* Presenting signs and symptoms in multiple myeloma: High percentages of stage III among patients without apparent myeloma-associated symptoms. *Ann Hematol* 1995;**70**:149–52. doi:10.1007/s002770050048

16 Greipp PR. International Staging System for Multiple Myeloma. *J Clin Oncol* 2005;**23**:3412–20. doi:10.1200/JCO.2005.04.242

17 Olesen F, Hansen RP, Vedsted P. Delay in diagnosis: the experience in Denmark. *Br J Cancer* 2009;**101 Suppl**:S5–8. doi:10.1038/sj.bjc.6605383

18 Weller D, Vedsted P, Rubin G, *et al.* The Aarhus statement: improving design and reporting of studies on early cancer diagnosis. *Br J Cancer* 2012;**106**:1262–7. doi:10.1038/bjc.2012.68

19 Neal RD, Tharmanathan P, France B, *et al.* Is increased time to diagnosis and treatment in symptomatic cancer associated with poorer outcomes? Systematic review. *Br J Cancer* 2015;**112 Suppl**:S92-107. doi:10.1038/bjc.2015.48

20 Elliss-Brookes L, McPhail S, Ives A, *et al.* Routes to diagnosis for cancer - determining the patient journey using multiple routine data sets. *Br J Cancer* 2012;**107**:1220–6. doi:10.1038/bjc.2012.408

21 Keeble S, Abel GA, Saunders CL, *et al.* Variation in promptness of presentation among 10,297 patients subsequently diagnosed with one of 18 cancers: Evidence from a National Audit of Cancer Diagnosis in Primary Care. *Int J Cancer* 2014;**135**:1220–8. doi:10.1002/ijc.28763

22 Howell DA, Warburton F, Ramirez A-J, *et al.* Risk factors and time to symptomatic presentation in leukaemia, lymphoma and myeloma. *Br J Cancer* 2015;**113**:1114–20. doi:10.1038/bjc.2015.311

23 Li N, Lv Y, Zeng H, *et al.* Renal impairment in multiple myeloma: Presenting features in different departments. *J Huazhong Univ Sci Technol [Medical Sci* 2012;**32**:65–8. doi:10.1007/s11596-012-0011-0

24 Din NU, Ukoumunne OC, Rubin G, *et al.* Age and Gender Variations in Cancer Diagnostic Intervals in 15 Cancers: Analysis of Data from the UK Clinical Practice Research Datalink. *PLoS One* 2015;**10**:e0127717. doi:10.1371/journal.pone.0127717

25 Neal RD, Din NU, Hamilton W, *et al.* Comparison of cancer diagnostic intervals before and after implementation of NICE guidelines: analysis of data from the UK General Practice Research Database. *Br J Cancer* 2014;**110**:584–92. doi:10.1038/bjc.2013.791

26 Lyratzopoulos G, Abel G a, McPhail S, *et al.* Measures of promptness of cancer diagnosis in primary care: secondary analysis of national audit data on patients with 18 common and rarer cancers. *Br J Cancer* 2013;**108**:1–5. doi:10.1038/bjc.2013.1

27 Lyratzopoulos G, Saunders CL, Abel GA, *et al.* The relative length of the patient and the primary care interval in patients with 28 common and rarer cancers. *Br J Cancer* 2015;**112 Suppl**:S35-40. doi:10.1038/bjc.2015.40

For peer review only - http://bmjopen.bmj.com/site/about/guidelines.xhtml

28 Swann R, McPhail S, Witt J, *et al.* Diagnosing cancer in primary care: results from the National Cancer Diagnosis Audit. *Br J Gen Pract* 2018;**68**:e63–72. doi:10.3399/bjgp17X694169

29 Varga G, Mikala G, Andrikovics H, *et al.* [How long does a myeloma patient currently wait for the diagnosis in Hungary?]. *Orv Hetil* 2014;**155**:1538–43. doi:10.1556/OH.2014.30000

30 Goldschmidt N, Zamir L, Poperno A, *et al.* Presenting Signs of Multiple Myeloma and the Effect of Diagnostic Delay on the Prognosis. *J Am Board Fam Med* 2016;**29**:702–9. doi:10.3122/jabfm.2016.06.150393

31 Howell DA, Smith AG, Jack A, *et al.* Time-to-diagnosis and symptoms of myeloma, lymphomas and leukaemias: a report from the Haematological Malignancy Research Network. *BMC Hematol* 2013;**13**:9. doi:10.1186/2052-1839-13-9

32 RCGP, Royal College of GPs. National Audit of Cancer Diagnosis in Primary Care. *Networks* 2011.

33 Corner J, Hopkinson J, Fitzsimmons D, *et al.* Is late diagnosis of lung cancer inevitable ? Interview study of patients' recollections of symptoms before diagnosis. 2005;:314–9. doi:10.1136/thx.2004.029264

34 Rubin GP, Saunders CL, Abel GA, *et al.* Impact of investigations in general practice on timeliness of referral for patients subsequently diagnosed with cancer : analysis of national primary care audit data. *Br J Cancer* 2015;**112**:676–87. doi:10.1038/bjc.2014.634

35 Howell D, Smith A, Appleton S, *et al.* Multiple myeloma : routes to diagnosis , clinical characteristics and survival – findings from a UK population-based study. 2017;:1–5. doi:10.1111/bjh.14513

36 Redaniel MT, Martin RM, Ridd MJ, *et al.* Diagnostic intervals and its association with breast, prostate, lung and colorectal cancer survival in England: Historical cohort study using the Clinical Practice Research Datalink. *PLoS One* 2015;**10**:1–17. doi:10.1371/journal.pone.0126608

37 Barrett J, Hamilton W. Pathways to the diagnosis of lung cancer in the UK: a cohort study. *BMC Fam Pract* 2008;**9**:31. doi:10.1186/1471-2296-9-31

38 Jensen H, Tørring ML, Olesen F, *et al.* Cancer suspicion in general practice , urgent referral and time to diagnosis : a population-based GP survey and registry study. 2014.

39 Ades AE, Biswas M, Welton NJ, *et al.* Symptom lead time distribution in lung cancer: Natural history and prospects for early diagnosis. *Int J Epidemiol* 2014;**43**:1865–73. doi:10.1093/ije/dyu174

For peer review only - http://bmjopen.bmj.com/site/about/guidelines.xhtml

Table 1: Study characteristics

Study design	Study period	Population characteristics (age, gender)	Myeloma patients (Total)	Outcome measure (Interval)
**Friese et al 2009, (USA)** [8]				
Retrospective analysis	1992-2002	mean age 76.3<br>46% males	3831	Diagnostic
**Howell et al 2013, (UK)** [31]				
Survey	2004-2011	Median age 69.9<br>66.9% males	341	Patient<br>Diagnostic<br>Time to diagnosis
**Lyratzopoulos et al 2013, (UK)** [26]				
Audit data	2009-2010	Not reported	176	Primary Care
**Varga et al 2014, (Hungary)** [29]				
Retrospective analysis	Not reported	median age 60<br>50% males	193	Diagnostic
**Neal et al 2014, (UK)** [25]				
Retrospective analysis	2001-2002	mean age 72<br>53% males	221	Diagnostic
**Din et al 2015, (UK)** [24]				
Retrospective analysis	2007-2010	median age 72<br>56% males	500	Diagnostic
**Lyratzopoulos et al 2015, (UK)** [27]				
Audit data	2009-2010	Not reported	124	Patient
**Goldscbmidt et al 2016, (Israel)** [30]				
Retrospective analysis	2002-2011	median age 63<br>53% males	107*	Diagnostic
**Swann et al 2017, (UK)** [28]				
Audit data	2014	Not reported	202	Primary<br>Diagnostic
*The total sample size for this study was 110 patients out of which 7 were diagnosed with plasmacytoma. The analysis was conducted on 107 patients that had complete data				

For peer review only - http://bmjopen.bmj.com/site/about/guidelines.xhtml

Table 2: Symptoms and date definitions

Symptoms used	Onset of first symptom	Date of first Presentation in healthcare services	Date of first referral	Date of diagnosis
**Friese et al 2009, (USA)**				
Anaemia<br>Packed red blood cell transfusion<br>Back pain	N/A	1 year before diagnosis	N/A	SEER cancer diagnosis date
**Howell et al 2013, (UK)**				
Tiredness<br>Pain<br>Shortness of breath<br>Infections<br>Joint problems/Fractures<br>Stomach/bowel symptoms<br>Other	Patient reported	Patient reported	N/A	Date provided by the Haematological Malignancy Diagnostic service
**Lyratzopoulos et al 2013, (UK)**				
Not reported	Estimated based on patient's clinical records.	2 years before diagnosis	Date that the referral letter was sent	Clinical records and hospital correspondence
**Varga et al 2014, (Hungary)**				
Bone symptoms<br>Anaemia<br>Renal failure<br>General symptoms<br>Other<br>Tumour presence<br>Metastatic bone disease	N/A	3 years before diagnosis	N/A	Tertiary haematology centre
**Neal et al 2014, (UK)**				
Bleeding<br>Bone Pain<br>Bruising<br>Anaemia<br>Fatigue<br>Anorexia<br>Weight loss	N/A	1 year before diagnosis	N/A	First occurrence of a myeloma Read Code in the patient's record in CPRD database.
**Din et al 2015, (UK)**				
Bleeding<br>Bone Pain<br>Bruising<br>Anaemia<br>Fatigue<br>Anorexia<br>Weight loss	N/A	1 year before diagnosis	N/A	First occurrence of a myeloma Read Code in the patient's record in CPRD database.
**Lyratzopoulos et al 2015, (UK)**				
Not reported	N/A	2 years before diagnosis	Date that the referral letter was sent	Clinical records and hospital correspondence

For peer review only
For peer review only - http://bmjopen.bmj.com/site/about/guidelines.xhtml

**Goldscbmidt et al 2016, (Israel)***				
Pain (back, cervical spine, musculoskeletal, non-specific)<br>Infection<br>Weight loss<br>Fatigue<br>Peripheral edema<br>Constipation<br>Presyncope<br>Syncope<br>Dizziness	N/A	2 years before diagnosis	N/A	Israel National Cancer Registry
**Swann et al 2017, (UK)**				
Not reported	NA	2 years before diagnosis	Date that the referral letter was sent	Hospital Episode Statistics (HES)

For peer review only

Table 3: Length of intervals

Percentile	N	25th	50th	75th
**Patient Interval**				
Howell et al 2013	341	1	31	122
Lyratzopoulos et al 2015	124	0	13.5	31
Weighted estimate	465	0.7	26.3	97.7
**Primary care Interval**				
Lyratzopoulos et al 2013	176	5	21	55
Swann et al 2017	150	4.2	23.5	56.8
Weighted estimate	326	4.6	21.6	55.8
**Referral to Diagnosis interval**				
No papers reporting this interval				
**Diagnostic interval**				
Friese et al 2009	3831	27	99	252
Howell et al 2013	341	34	83	167
Varga et al, 2014	193	88	125	230
Neal et al, 2014	221	56	144	264
Din et al 2015	500	54	149	263
Goldscbmidt et al 2016	107	NR*	341	NR*
Swann et al 2017	202	24	53.5	107.5
Weighted estimate	5395	33.3	108.6	241.7
**Time to diagnosis**				
Howell et al, 2013	341	84	163	306

*Not reported

For peer review only - http://bmjopen.bmj.com/site/about/guidelines.xhtml

Titles and legends to figures

Figure 1

Title: Outcome definition

Figure 2

Title: Study selection flowchart

Figure 3

Title: Distribution of the intervals

Legend: Each circle corresponds to one study and the size is proportional to the total sample size. The blue diamond corresponds to the weighted estimate. For intervals with only one study (time to diagnosis) no weighted estimates were calculated. Y-axis corresponds to 1-Probability (interval > number of days) i.e. 0.25 corresponds to the 75th percentile and 0.75 to the 25th percentile.

For peer review only

For peer review only - http://bmjopen.bmj.com/site/about/guidelines.xhtml

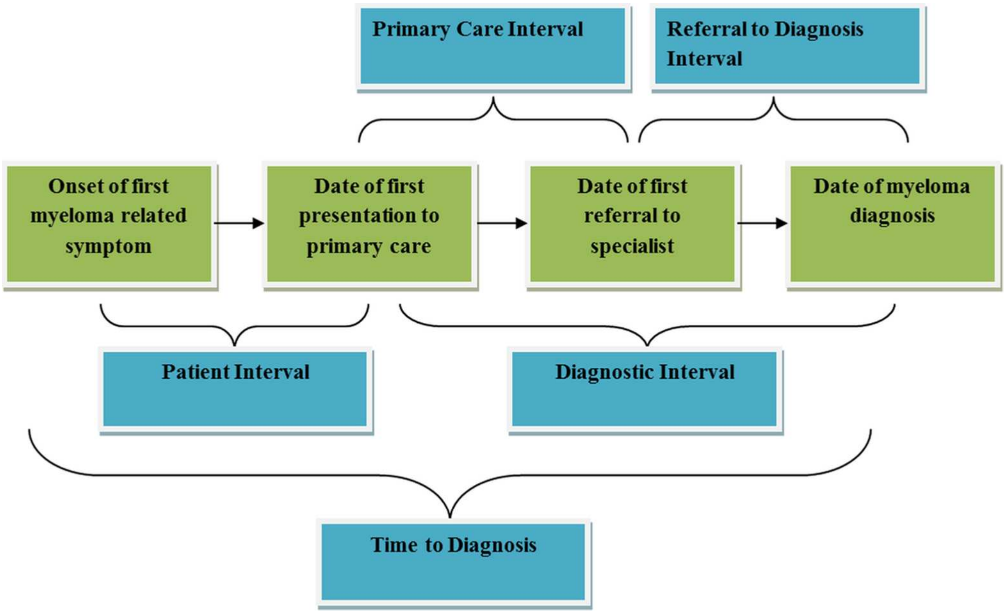

Figure 1: Outcome definition

108x65mm (300 x 300 DPI)

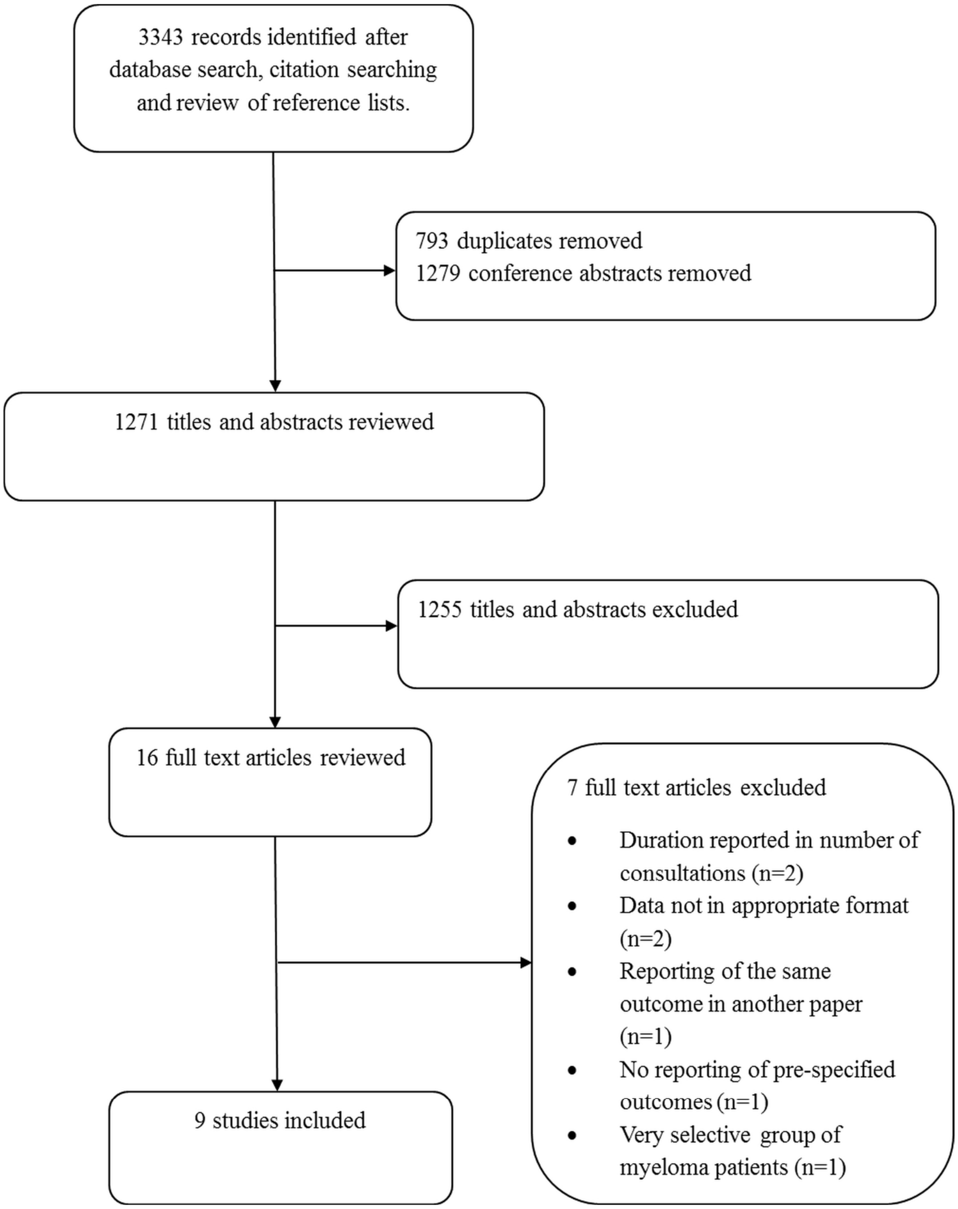

Figure 2: Study flowchart

214x270mm (300 x 300 DPI)

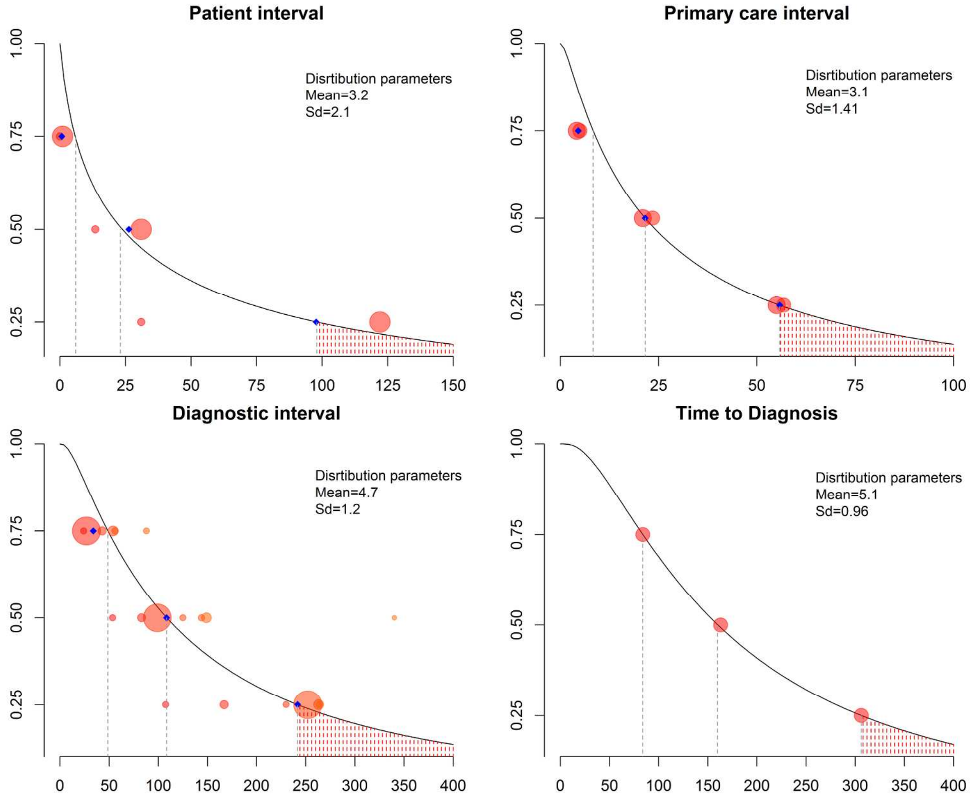

Figure 3: Distribution of the intervals. Each circle corresponds to one study and the size is proportional to the total sample size. The blue diamond corresponds to the weighted estimate. For intervals with only one study (time to diagnosis) no weighted estimates were calculated. Y-axis corresponds to 1-Probability (interval > number of days) i.e. 0.25 corresponds to the 75th percentile and 0.75 to the 25th percentile

237x189mm (300 x 300 DPI)

A1: Systematic review protocol

Title:

Delays observed in the pathway leading to the diagnosis of multiple myeloma - a systematic review

Background

Multiple myeloma is one of the most common haematological malignancies, with more than 4000 cases diagnosed annually. It is considered as one of the most difficult cancers to diagnose due the very non-specific nature of symptoms which might include bone pain, fatigue, dyspnoea, weight loss, repeated infections etc. 51% of the symptomatic myeloma patients have to visit their GP at least 3 times before they get a confirmed diagnosis of the disease and 38% of the patients are identified through emergency admissions (compared to 23% for the rest of the cancers)1. A study has suggested that a delayed diagnosis had a significant effect on disease-free survival 2 so an earlier diagnosis of the disease could potentially lead to fewer complications, a better prognosis and a better quality of life. The aim of this systematic review is to examine the published literature for diagnostic delay in multiple myeloma across the diagnostic pathway.

1. Lyratzopoulos, G., Neal, R. D., Barbiere, J. M., Rubin, G. P. & Abel, G. a. Variation in number of general practitioner consultations before hospital referral for cancer: Findings from the 2010 National Cancer Patient Experience Survey in England. Lancet Oncol. 13, 353–365 (2012).

2. Kariyawasan, C. C., Hughes, D. a., Jayatillake, M. M. & Mehta, a. B. Multiple myeloma: causes and consequences of delay in diagnosis. Qjm 100, 635–640 (2007).

Review questions/objectives:

Quantification of the time intervals that multiple myeloma patients experience from first symptom to confirmation of diagnosis. The time interval will include the patient intervals (onset of first symptom to help seeking), primary care interval (from first presentation to primary care until first referral), secondary care interval (first referral to diagnosis), diagnostic interval (first presentation to diagnosis) and the total interval (onset of symptoms to diagnosis).

Searches and eligibility criteria

A systematic literature search will be performed in MEDLINE and EMBASE. All articles that are quantifying any of the intervals mentioned above will be included. Articles on non-adults (<18 years) and on the asymptomatic form of the disease will be excluded.

Type of studies to be included:

Cross-sectional surveys, prospective patient studies and retrospective analysis of medical records which give a numerical measure of diagnostic delay in multiple myeloma. Only full text articles will be included in the review. Conference abstracts will be excluded.

Intervention(s)/exposure(s)

For peer review only

None

Comparator(s)/control

None

Primary outcomes

Clinical diagnostic interval (first presentation to final diagnosis)

Patient interval (symptom onset to first presentation)

Primary care interval (first presentation to first referral)

Secondary care interval (first referral to final diagnosis)

Total interval (symptom onset to final diagnosis)

Data extraction (selection and coding)

Two reviewers (CK and LA) will extract the data from the included studies in pre-specified forms. Disagreements will be resolved by consulting a third reviewer (AVB or JO). Variables that are going to be extracted from the papers include: Author, Year of study, Country, Study Design, sample size, initial symptoms and descriptive statistics for the interval under investigation (mean, Sd, median and IQR).

Risk of bias (quality assessment)

The methodological quality of the papers will be assessed by using the Aarhus checklist

Strategy for data synthesis

Mean, median and interquartile range will be extracted for the analysis but since time duration is usually not a normally distributed variable the median and IQR will be preferred. Delays that are reported in months will be transformed into days. In papers where the number of consultations is reported instead of a numeric value of the delay we will contact authors otherwise these papers will be excluded. We will try to combine our estimates in order to get an overall estimate of the delay observed for each interval.

Analysis of subgroups or subsets

Subgroup analysis will be conducted comparing studies with the highest risk of bias versus the rest.

Dissemination plans

This review will be published in a peer-reviewed journal and presented in relevant conferences

For peer review only

A2: Search strategy

1. Multiple Myeloma/
2. myeloma*.ti,ab.
3. 1 or 2
4. (time adj4 diagnos$).ti,ab.
5. (time adj4 consult$).ti,ab.
6. (time adj4 refer$).ti,ab.
7. (time adj4 present$).ti,ab.
8. 4 or 5 or 6 or 7
9. (delay$ adj4 diagnos$).ti,ab.
10. (delay$ adj4 consult$).ti,ab.
11. (delay$ adj4 refer$).ti,ab.
12. (delay$ adj4 present$).ti,ab.
13. (delay$ adj4 seek$).ti,ab.
14. (delay$ adj4 detect*).ti,ab.
15. 9 or 10 or 11 or 12 or 13 or 14
16. (interval adj4 consult$).ti,ab.
17. (interval adj4 consult$).ti,ab.
18. (interval adj4 refer$).ti,ab.
19. (interval adj4 present$).ti,ab.
20. 16 or 17 or 18 or 19
21. (late adj4 diagnosis).ti,ab.
22. (late adj4 detect*).ti,ab.
23. (late adj4 present$).ti,ab.
24. 21 or 22 or 23
25. diagnos$ delay$.ti,ab.
26. early diagnos$.ti,ab.
27. 8 or 15 or 20 or 24 or 25 or 26
28. 3 and 27

For peer review only

A3: Risk of bias summary

No / Yes / Not applicable	1	2	3	4	5	6	7	8	9	10	11	12	13	14	15	16	17	18	19	20
Friese et al. 2009	Yes	Yes	Not applicable	Not applicable	No	Not applicable	No	Yes	Yes	No	Not applicable	Not applicable	Not applicable	Not applicable	Not applicable	Not applicable	Not applicable	Not applicable	Not applicable	Yes
Howell et al. 2013	Yes	Yes	No	Yes	Yes	Not applicable	No	Yes	Yes	Yes	No	Yes	No	No	Yes	Yes	No	Yes	Not applicable	Not applicable
Lyratzopoulos et al. 2013	Yes	Yes	Not applicable	Not applicable	Yes	Yes	No	Yes	Yes	Yes	Not applicable	Not applicable	Not applicable	Not applicable	Not applicable	Not applicable	Not applicable	Not applicable	Not applicable	Yes
Varga et al. 2014	Yes	No	Not applicable	Not applicable	No	Not applicable	No	Yes	Yes	No	Not applicable	Not applicable	Not applicable	Not applicable	Not applicable	Not applicable	Not applicable	Not applicable	No	Not applicable
Neal et al. 2014	Yes	Yes	Not applicable	Not applicable	Yes	Not applicable	Yes	Yes	Yes	Yes	Not applicable	Not applicable	Not applicable	Not applicable	Not applicable	Not applicable	Not applicable	Not applicable	Not applicable	Yes
Din et al. 2015	Yes	Yes	Not applicable	Not applicable	Yes	Not applicable	Yes	Yes	Yes	Yes	Not applicable	Not applicable	Not applicable	Not applicable	Not applicable	Not applicable	Not applicable	Not applicable	Not applicable	Yes
Lyratzopoulos et al. 2015	Yes	Yes	Yes	Yes	Yes	Not applicable	No	Yes	Yes	Yes	Not applicable	Not applicable	Not applicable	Not applicable	Not applicable	Not applicable	Not applicable	Not applicable	Not applicable	Yes
Goldscbmidt et al. 2016	Yes	Yes	Not applicable	Not applicable	Yes	Not applicable	No	Yes	Yes	No	Not applicable	Not applicable	Not applicable	Not applicable	Not applicable	Not applicable	Not applicable	Not applicable	Yes	Not applicable
Swann et al. 2017	Yes	Yes	Not applicable	Not applicable	Yes	Yes	No	Yes	Yes	Yes	Not applicable	Not applicable	Not applicable	Not applicable	Not applicable	Not applicable	Not applicable	Not applicable	Yes	Not applicable

A4: Risk of bias graph

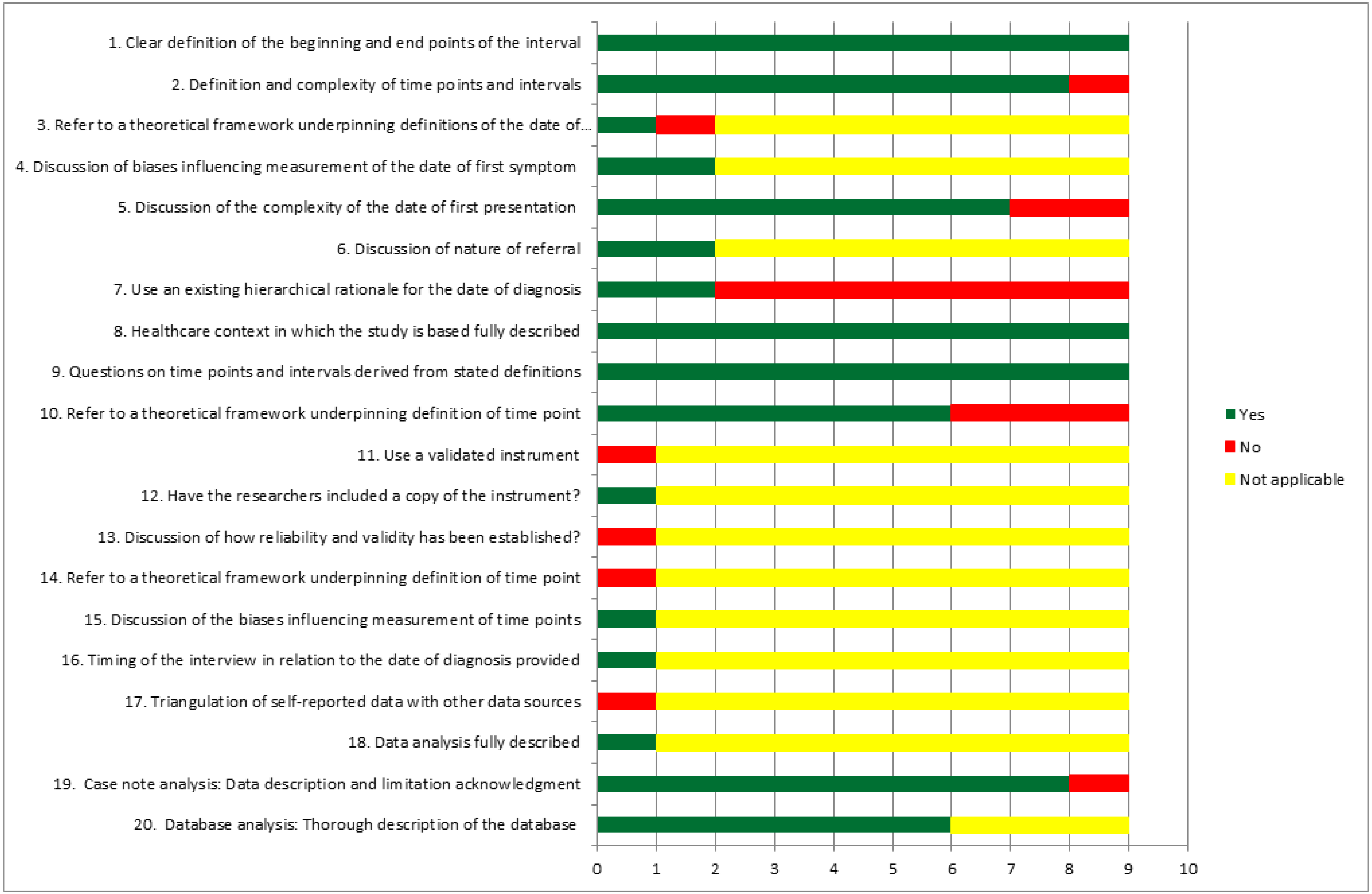

A5: Random effects meta-analysis combining means. For studies that were not reporting means they were approximated using the median and interquartile range.

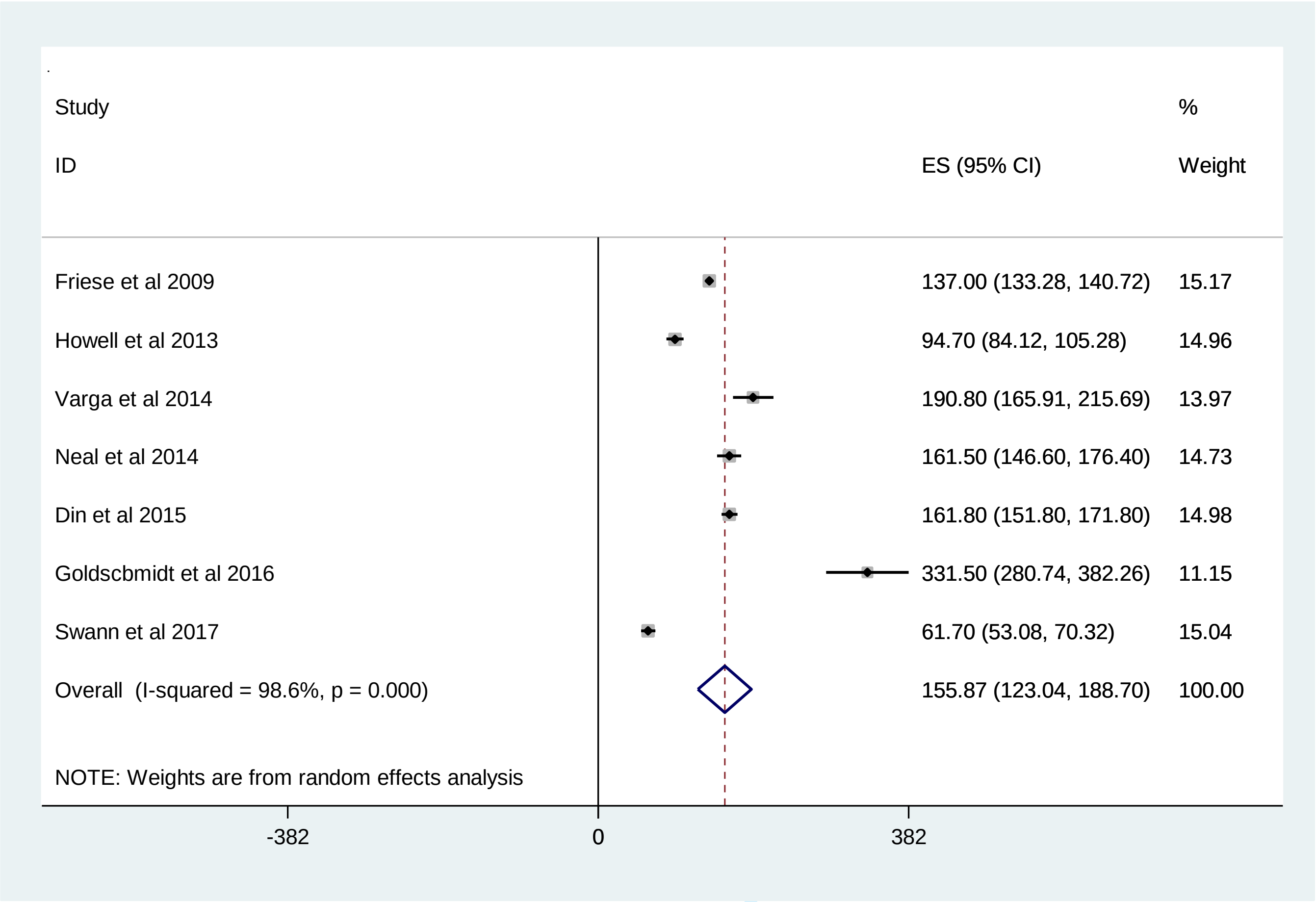

w only

PRISMA 2009 Checklist

Section/topic	#	Checklist item	Reported on page #
**TITLE**			
Title	1	Identify the report as a systematic review, meta-analysis, or both.	1
**ABSTRACT**			
Structured summary	2	Provide a structured summary including, as applicable: background; objectives; data sources; study eligibility criteria, participants, and interventions; study appraisal and synthesis methods; results; limitations; conclusions and implications of key findings; systematic review registration number.	2
**INTRODUCTION**			
Rationale	3	Describe the rationale for the review in the context of what is already known.	4-5
Objectives	4	Provide an explicit statement of questions being addressed with reference to participants, interventions, comparisons, outcomes, and study design (PICOS).	5-6
**METHODS**			
Protocol and registration	5	Indicate if a review protocol exists, if and where it can be accessed (e.g., Web address), and, if available, provide registration information including registration number.	5-6
Eligibility criteria	6	Specify study characteristics (e.g., PICOS, length of follow-up) and report characteristics (e.g., years considered, language, publication status) used as criteria for eligibility, giving rationale.	5-6
Information sources	7	Describe all information sources (e.g., databases with dates of coverage, contact with study authors to identify additional studies) in the search and date last searched.	5-6
Search	8	Present full electronic search strategy for at least one database, including any limits used, such that it could be repeated.	p.5 & Appendix
Study selection	9	State the process for selecting studies (i.e., screening, eligibility, included in systematic review, and, if applicable, included in the meta-analysis).	6
Data collection process	10	Describe method of data extraction from reports (e.g., piloted forms, independently, in duplicate) and any processes for obtaining and confirming data from investigators.	6
Data items	11	List and define all variables for which data were sought (e.g., PICOS, funding sources) and any assumptions and simplifications made.	6
Risk of bias in individual studies	12	Describe methods used for assessing risk of bias of individual studies (including specification of whether this was done at the study or outcome level), and how this information is to be used in any data synthesis.	6
Summary measures	13	State the principal summary measures (e.g., risk ratio, difference in means).	6
Synthesis of results	14	Describe the methods of handling data and combining results of studies, if done, including measures of consistency (e.g., I^2) for each meta-analysis.	6

For peer review only

PRISMA 2009 Checklist

Section/topic	#	Checklist item	Reported on page #
Risk of bias across studies	15	Specify any assessment of risk of bias that may affect the cumulative evidence (e.g., publication bias, selective reporting within studies).	6-7
Additional analyses	16	Describe methods of additional analyses (e.g., sensitivity or subgroup analyses, meta-regression), if done, indicating which were pre-specified.	6-7
**RESULTS**			
Study selection	17	Give numbers of studies screened, assessed for eligibility, and included in the review, with reasons for exclusions at each stage, ideally with a flow diagram.	7 (figure 1)
Study characteristics	18	For each study, present characteristics for which data were extracted (e.g., study size, PICOS, follow-up period) and provide the citations.	7 (table 1)
Risk of bias within studies	19	Present data on risk of bias of each study and, if available, any outcome level assessment (see item 12).	Appendix
Results of individual studies	20	For all outcomes considered (benefits or harms), present, for each study: (a) simple summary data for each intervention group (b) effect estimates and confidence intervals, ideally with a forest plot.	p. 7-9 table 2, figure 3
Synthesis of results	21	Present results of each meta-analysis done, including confidence intervals and measures of consistency.	p. 7-9 table 2, figure 3
Risk of bias across studies	22	Present results of any assessment of risk of bias across studies (see Item 15).	8-9 appendix
Additional analysis	23	Give results of additional analyses, if done (e.g., sensitivity or subgroup analyses, meta-regression [see Item 16]).	9
**DISCUSSION**			
Summary of evidence	24	Summarize the main findings including the strength of evidence for each main outcome; consider their relevance to key groups (e.g., healthcare providers, users, and policy makers).	9-10
Limitations	25	Discuss limitations at study and outcome level (e.g., risk of bias), and at review-level (e.g., incomplete retrieval of identified research, reporting bias).	10-12
Conclusions	26	Provide a general interpretation of the results in the context of other evidence, and implications for future research.	10-13
**FUNDING**			

For peer review only

PRISMA 2009 Checklist

Funding	27	Describe sources of funding for the systematic review and other support (e.g., supply of data); role of funders for the systematic review.	14

From: Moher D, Liberati A, Tetzlaff J, Altman DG, The PRISMA Group (2009). Preferred Reporting Items for Systematic Reviews and Meta-Analyses: The PRISMA Statement. PLoS Med 6(7): e1000097. doi:10.1371/journal.pmed1000097

For more information, visit: **www.prisma-statement.org**.

For peer review only

MOOSE Checklist for Meta-analyses of Observational Studies

Item No	Recommendation	Reported on Page No
Reporting of background should include		
1	Problem definition	P4
2	Hypothesis statement	P5
3	Description of study outcome(s)	P5
4	Type of exposure or intervention used	NA
5	Type of study designs used	P5
6	Study population	P5
Reporting of search strategy should include		
7	Qualifications of searchers (eg, librarians and investigators)	P5
8	Search strategy, including time period included in the synthesis and key words	P5 & appendix
9	Effort to include all available studies, including contact with authors	P6
10	Databases and registries searched	P5
11	Search software used, name and version, including special features used (eg, explosion)	P5 & appendix
12	Use of hand searching (eg, reference lists of obtained articles)	NA
13	List of citations located and those excluded, including justification	P7/F2
14	Method of addressing articles published in languages other than English	P5
15	Method of handling abstracts and unpublished studies	P5
16	Description of any contact with authors	P6
Reporting of methods should include		
17	Description of relevance or appropriateness of studies assembled for assessing the hypothesis to be tested	P5
18	Rationale for the selection and coding of data (eg, sound clinical principles or convenience)	P5-6
19	Documentation of how data were classified and coded (eg, multiple raters, blinding and interrater reliability)	P5-6
20	Assessment of confounding (eg, comparability of cases and controls in studies where appropriate)	NA
21	Assessment of study quality, including blinding of quality assessors, stratification or regression on possible predictors of study results	P 6-7
22	Assessment of heterogeneity	P 7-8, 10
23	Description of statistical methods (eg, complete description of fixed or random effects models, justification of whether the chosen models account for predictors of study results, dose-response models, or cumulative meta-analysis) in sufficient detail to be replicated	P 7-8
24	Provision of appropriate tables and graphics	T1,T2,T3, F1, F2, F3
Reporting of results should include		
25	Graphic summarizing individual study estimates and overall estimate	T2, F3
26	Table giving descriptive information for each study included	T1
27	Results of sensitivity testing (eg, subgroup analysis)	P9, 11
28	Indication of statistical uncertainty of findings	P10

For peer review only

For peer review only - http://bmjopen.bmj.com/site/about/guidelines.xhtml

Item No	Recommendation	Reported on Page No
Reporting of discussion should include		
29	Quantitative assessment of bias (eg, publication bias)	P8
30	Justification for exclusion (eg, exclusion of non-English language citations)	NA
31	Assessment of quality of included studies	P8, appendix
Reporting of conclusions should include		
32	Consideration of alternative explanations for observed results	P10-11
33	Generalization of the conclusions (ie, appropriate for the data presented and within the domain of the literature review)	P11-12
34	Guidelines for future research	P12
35	Disclosure of funding source	P14

From: Stroup DF, Berlin JA, Morton SC, et al, for the Meta-analysis Of Observational Studies in Epidemiology (MOOSE) Group. Meta-analysis of Observational Studies in Epidemiology. A Proposal for Reporting. *JAMA*. 2000;283(15):2008-2012. doi: 10.1001/jama.283.15.2008.

Transcribed from the original paper within the NEUROSURGERY® Editorial Office, Atlanta, GA, United Sates. August 2012.

For peer review only
